# Development and topographical organization of projections from the hippocampus and parahippocampus to the retrosplenial cortex

**DOI:** 10.1111/ejn.14395

**Published:** 2019-03-25

**Authors:** Kamilla G. Haugland, Jørgen Sugar, Menno P. Witter

**Affiliations:** ^1^ Kavli Institute for Systems Neuroscience Centre for Neural Computation, Egil and Pauline Braathen and Fred Kavli Center for Cortical Microcircuits NTNU Norwegian University for Science and Technology Trondheim Norway; ^2^ Present address: Department of Clinical Medicine University of Tromsø—The Arctic University of Norway Tromsø Norway

**Keywords:** connectivity, entorhinal cortex, parasubiculum, presubiculum, subiculum

## Abstract

The rat hippocampal formation (HF), parahippocampal region (PHR), and retrosplenial cortex (RSC) play critical roles in spatial processing. These regions are interconnected, and functionally dependent. The neuronal networks mediating this reciprocal dependency are largely unknown. Establishing the developmental timing of network formation will help to understand the emergence of this dependency. We questioned whether the long‐range outputs from HF‐PHR to RSC in Long Evans rats develop during the same time periods as previously reported for the intrinsic HF‐PHR connectivity and the projections from RSC to HF‐PHR. The results of a series of retrograde and anterograde tracing experiments in rats of different postnatal ages show that the postnatal projections from HF‐PHR to RSC display low densities around birth, but develop during the first postnatal week, reaching adult‐like densities around the time of eye‐opening. Developing projections display a topographical organization similar to adult projections. We conclude that the long‐range projections from HF‐PHR to RSC develop in parallel with the intrinsic circuitry of HF‐PHR and the projections of RSC to HF‐PHR.

**Abbreviations**
BDA: biotinylated dextran amine
EC: entorhinal cortex
FB: fast blue
HF: hippocampal formation
LEC: lateral entorhinal cortex
MEC: medial entorhinal cortex
PaS: parasubiculum
PB: phosphate buffer
PER: perirhinal cortex
PHR: parahippocampal region
POR: postrhinal cortex
P: postnatal
PrS: presubiculum
RSC: retrosplenial cortex
SUB: subiculum

## 1 INTRODUCTION

The hippocampal formation (HF) and the parahippocampal region (PHR) play critical roles in spatial processing (Jarrard, 1978; Maguire, Nannery, & Spiers, 2006). In both regions, spatially modulated neurons are found. Place cells, located in HF, fire at specific locations in the environment (O'Keefe & Dostrovsky, 1971), whereas grid cells, head direction cells, border cells, and speed cells have been reported in various subdivisions of PHR, such as the medial entorhinal cortex (MEC), presubiculum (PrS), and parasubiculum (PaS; Taube, Muller, & Ranck, 1990; Fyhn, Molden, Witter, Moser, & Moser, 2004; Solstad, Boccara, Kropff, Moser, & Moser, 2008; Boccara et al., 2010; Kropff, Carmichael, Moser, & Moser, 2015). It is currently thought that the HF‐PHR interaction is necessary to maintain proper place cell and grid cell modulation (Bonnevie et al., 2013; Renno‐Costa & Tort, 2017; Solstad, Moser, & Einevoll, 2006). In the classical model of HF‐PHR circuitry, neurons in superficial layers of EC are the main source of cortical input to HF. Output from the HF originates in Cornu Ammonis fields CA2, and CA1, and the subiculum (SUB), targeting several subcortical and cortical structures including the deep layers of EC as well as PrS and PaS. Neurons in the deep layers of EC originate a main output of the HF‐PHR, reaching several subcortical and cortical structures (Cappaert, Van Strien, & Witter, 2015).

Several of HF and PHR subdivisions, in particular, SUB, PrS, PaS, and MEC share connectivity with the retrosplenial cortex (RSC; Finch, Derian, & Babb, 1984; Agster & Burwell, 2009; Honda, Furuta, Kaneko, Shibata, & Sasaki, 2011; Honda & Ishizuka, 2015). Located dorsal to HF and PHR, RSC forms the most caudal portion of the cingulate cortex, and has been suggested to form a continuum with PrS in the monkey (Berger, Alvarez, & Pelaprat, 1997). A close functional relationship between HF‐PHR and RSC is evident as RSC also contains spatially modulated neurons (Alexander & Nitz, 2015; Cho & Sharp, 2001; Mao, Kandler, McNaughton, & Bonin, 2017). Malfunction of the RSC results in diminished spatial memory, like the outcome of a dysfunctional HF‐PHR (Iaria, Bogod, Fox, & Barton, 2009; Maguire, 2001; Sutherland, Whishaw, & Kolb, 1988). Given that both HF‐PHR and RSC are important for spatial cognitive functions, it has been suggested that these areas are reciprocally dependent on each other for proper processing of spatial information (Ranganath & Ritchey, 2012; Vann, Aggleton, & Maguire, 2009). However, the neuronal networks and mechanisms mediating this reciprocal dependency are largely unknown.

In recent years, we and others have used analyses of brain development as a way to study the functional interactions and respective dependencies between neurons and networks in the HF‐PHR (Bjerknes, Langston, Kruge, Moser, & Moser, 2015; Canto, Koganezawa, & Witter, 2011; Donato, Jacobsen, Moser, & Moser, 2017; Langston et al., 2010; Muessig, Hauser, Wills, & Cacucci, 2015; O'Reilly, Gulden Dahl, Ulsaker Kruge, & Witter, 2013; O'Reilly et al., 2014; Sugar & Witter, 2016; Wills, Cacucci, Burgess, & O'Keefe, 2010). Our data suggest that the HF‐EC interconnectivity and long‐range inputs to HF‐PHR start to develop around birth, and display adult‐like topographical organizations before eye‐opening and before the animal starts active exploration of the environment (O'Reilly et al., 2013, 2014; Sugar & Witter, 2016). In view of the close functional relationship between HF‐PHR and RSC, we aimed to investigate whether long‐range outputs from HF‐PHR develop during the same time periods as the intrinsic HF‐PHR connectivity and long‐range inputs to the region. In this study, we describe the postnatal development of HF‐PHR projections to RSC based on a series of retrograde and anterograde tracing experiments in rats of different postnatal ages.

## 2 MATERIALS AND METHODS

### 2.1 Surgeries and perfusion

In this study, we used a total of 69 female and male Long Evans rats aged between postnatal day 0 (P0) and 15 (P15). The pups were bred in‐house and housed in enriched cages together with their parents and littermates. Cages were checked for pups every morning and evening, and the day pups were observed was considered P0. In the paper, we describe data related to the age of the animal, meaning data derived from animals perfused at that particular day. Litters with more than 10 pups were culled to 10 at P0 or P1 to avoid unnecessary stress for the animals. The animals lived in a controlled environment (22 ± 1°C; humidity 60%; lights on from 8:00 p.m. to 8:00 a.m.). Food and water were available ad libitum. The experimental protocols followed the European Communities Council Directive and the Norwegian Experiments on Animals Act and local directives of the responsible veterinarian at the Norwegian University of Science and Technology.

All surgeries were conducted under isoflurane gas anesthesia. Animals were placed in an induction chamber and fully anesthetized before they were moved to a stereotaxic frame where the head was fixed using a neonatal mask and mouthpiece (model 973‐B; Kopf, Tujunga, CA, USA) and zygoma ear cups (model 921; Kopf). Before incision, the skin was disinfected with 2% iodine in 65% ethanol, and as a local analgesic, Bupivacain (0.2 ml per 100 g bodyweight of a 0.5 mg/ml solution; Marcain, Astra Zeneca, London, UK) was injected subcutaneously at the place of incision. The skin was opened with a small‐sized and sharp tipped scissor. After incision, the mouthpiece and ear cups were adjusted so that bregma and lambda were aligned horizontally. The bone over the injection site and over the posterior extreme of the sagittal sinus was then removed. The exact place of injection was determined using the junction of the transverse and sagittal sinus as a reference for the anteroposterior coordinate, the lateral edge of the midsagital sinus as a reference for the mediolateral coordinate, and the level of the dura as a reference for the dorsoventral coordinate. The retrograde tracers Fast Blue (FB; 1% in 0.125 M PB, EMS Chemie, Domat, Switzerland), Fluoro Gold (2.5% in H_2_0, Fluorochrome, Denver, CO, USA), DiI (1.5% in EtOH, Paisley, UK), IX retrobeads (Red and Green, Lumafluor, Durham, NC), FluoSpheres 505/515 (0.04 μm, 5%, F8795 Thermo Fischer Scientific [TMS], Waltham, MA, USA), and FluoSpheres 580/605 (0.02 μm, 2%, F8786, TMS) were pressure injected at the identified coordinates. Before injection, the dura was punctured, and glass micropipettes with an outer tip‐diameter of 30–60 μm (30‐0044, Harvard Apparatus, MA. Pulled with a PP‐830 puller, Narishige, Japan) or Hamilton syringes (1 μl, 25 ga, 7001, Sigma‐Aldrich, St. Louis, MO, USA) were used to deliver the tracer. For the majority of the experiments, we used glass micropipettes connected to a pneumatic pump. To avoid dehydration during surgery, appropriate amounts of sterile saline (room temperature) were administered subcutaneously. Animals received carprofen during surgery, which acted as an analgesic during surgery and the first 24 hr after surgery (1 ml per 100 g bodyweight of a 0.5 mg/ml solution; Rimadyl, Pfizer, NY, USA). After surgery, the incision was sutured and the pups were allowed to recover under a heating lamp. When fully awake, the pups were returned to maternal care until the time of kill. In addition to the retrograde tracer injections, we injected, in a subset of animals (*n* = 11), the anterograde tracer biotinylated dextran amine (BDA; 5% in phosphate buffer (PB; 0.125 M in H_2_0; pH 7.4), 10,000 MW, D1956, Invitrogen, Eugene, OR, USA) into SUB and PHR. The tracer was delivered through micropipettes with an outer tip‐diameter of 20–25 μm, using iontophoresis (4–6 μA, alternating currents, 6 s on/6 s off, for 5–15 min current source 51595; Stoelting, Wood Dale, IL, USA).

We euthanized the animals 18–30 hr after surgery under terminal anesthesia with isoflurane. The thorax was opened with a small‐sized scissor and cold Ringer's solution was perfused through the body by inserting the needle in the left ventricle and opening the right auricle. When the liver turned pale (~30 s), the perfusion solution was changed to a 4% solution of freshly depolymerized paraformaldehyde in PB (pH 7.4). In the case of P0–P2 animals, 0.1% glutaraldehyde was added to the fixative. The brain was removed from the skull and postfixed overnight at 4°C in the same fixative. Twenty‐four hours after perfusion, the brains were transferred to PB containing 2% DMSO (VWR) and 20% glycerol (VWR).

### 2.2 Tissue processing

Brains were cut on a freezing microtome (HM‐430 Thermo Scientific, Waltham, MA) in 40 or 50 μm thick horizontal or sagittal sections. Depending on the age of the animal, sections were collected in 4–6 equally spaced series. For experiments using retrograde tracers, two of the series were mounted directly on superfrost slides (Thermo Scientific). For experiments using anterograde tracers, one of the series was mounted directly on superfrost slides. Another series was stained to visualize the anterograde tracer biotinylated dextran amine as previously described (O'Reilly et al., 2013). The remaining sections were collected in vials containing 2% DMSO and 20% glycerol in PB and stored at −20°C until further processing. One of the mounted series was Nissl stained. The sections were dehydrated in ethanol, cleared in xylene (VWR), and rehydrated before placed in cresyl violet (0.1% in water, C5042, Sigma) for 2–6 min. Subsequently, the sections were rinsed quickly in water, before placing them in 50% ethanol containing acetic acid to differentiate the staining. The stained sections were dehydrated in ethanol, cleared in xylene and finally coverslipped with Entellan (107961, Merck, Darmstadt, Germany).

In experiments with retrograde tracers, the second mounted series was cleared in toluene and coverslipped with Entellan for microscopic visualization using fluorescence illumination at the appropriate excitation wavelengths (Zeiss Axio Imager M1/2). Digital images of successful injections, retrogradely labeled neurons in PHR or Nissl‐stained tissue were obtained using a slide scanner equipped for either brightfield or fluorescence imaging (Zeiss Mirax Midi; objective 20X; NA 0.8). Next, two independent persons assessed all digital images. In the few cases when there were discrepancies between the two observers, we returned to the actual sections to resolve any discrepancies. For illustrative purposes, images of sections were exported using Panoramic Viewer software (3DHistech, Budapest, Hungary) and processed in Adobe Photoshop and Illustrator (CS6, Adobe Systems, San Jose, CA, USA).

### 2.3 Location of injections

**Figure 1:**
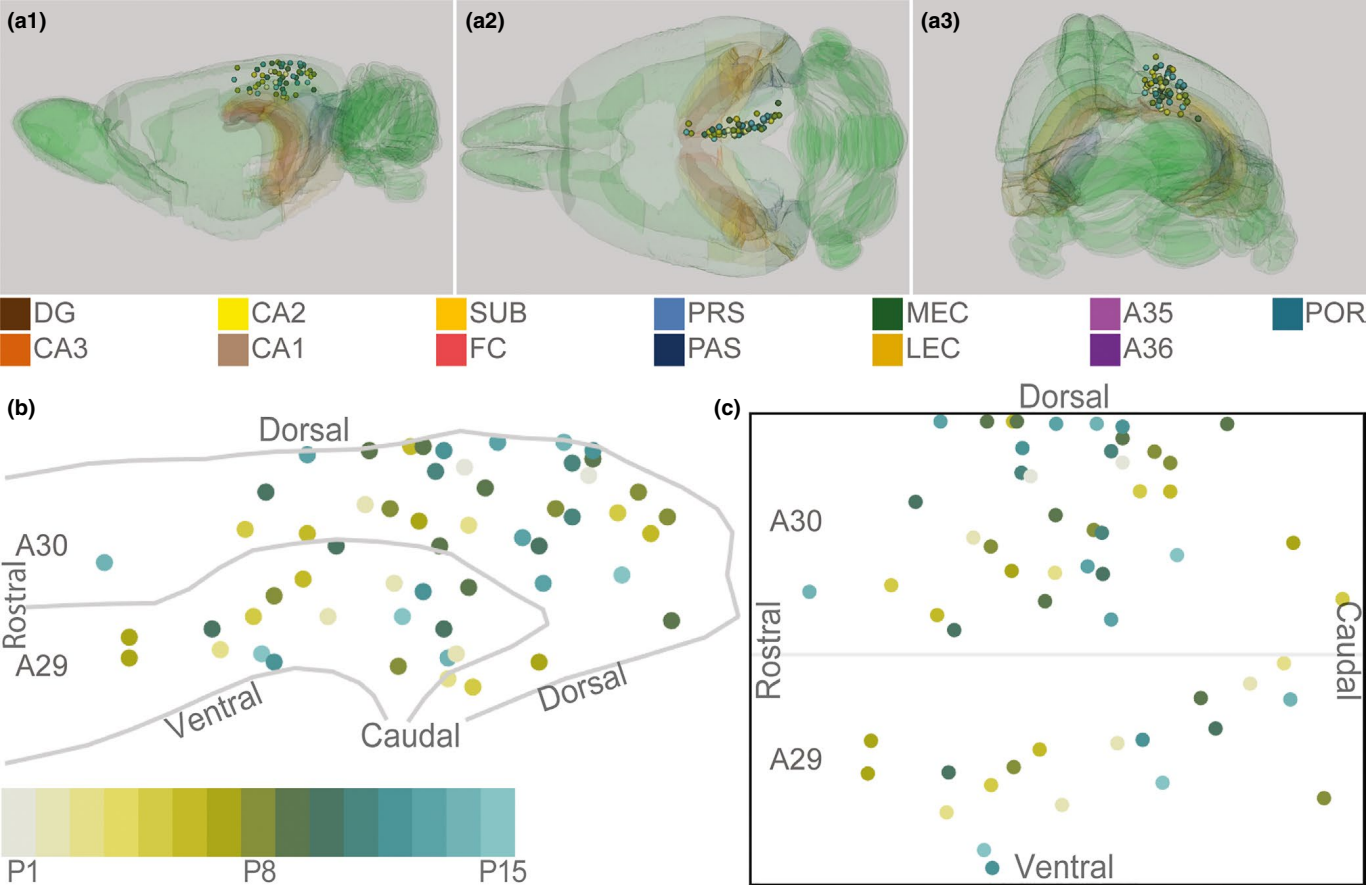
Schematic representation of injections in RSC. (a) The location of the center of each injection was normalized to a standard 3D atlas of the rat brain (Waxhom space, http://software.incf.org; Papp et al., 2014). Sagittal (a1), dorsal (a2), and caudal view (a3) of the 3D brain with the center of each injection (colored spheres). Para(hippocampal) structures are color‐coded, and the rest of the brain is colored green. In all panels, each injection is color‐coded according to the bottom left color scheme; light gray colored injections represent injections in pups aged P1, green colored injections represent injections in pups aged P8, whereas cyan colored injections represent injections in pups aged close to P15. (b) Midsagital view of the center of the injections projected to the pial surface. The mediolateral position was disregarded to allow the injections to be plotted in 2D. Gray lines depict dorsal and ventral borders of RSC and the border between A29 and A30 of RSC. (c) Flatmap of the injections (see methods for details). The 3D RSC was converted to a 2D normalized flatmap to represent the relative rostrocaudal and dorsoventral positions of all injections. The figure is oriented with rostral RSC (left), caudal RSC (right), dorsal RSC (top) ventral RSC (bottom) to each of the sides of the rectangle. Gray line depicts the border between A29 and A30 in RSC. [Colour figure can be viewed at wileyonlinelibrary.com]

**Figure 2:**
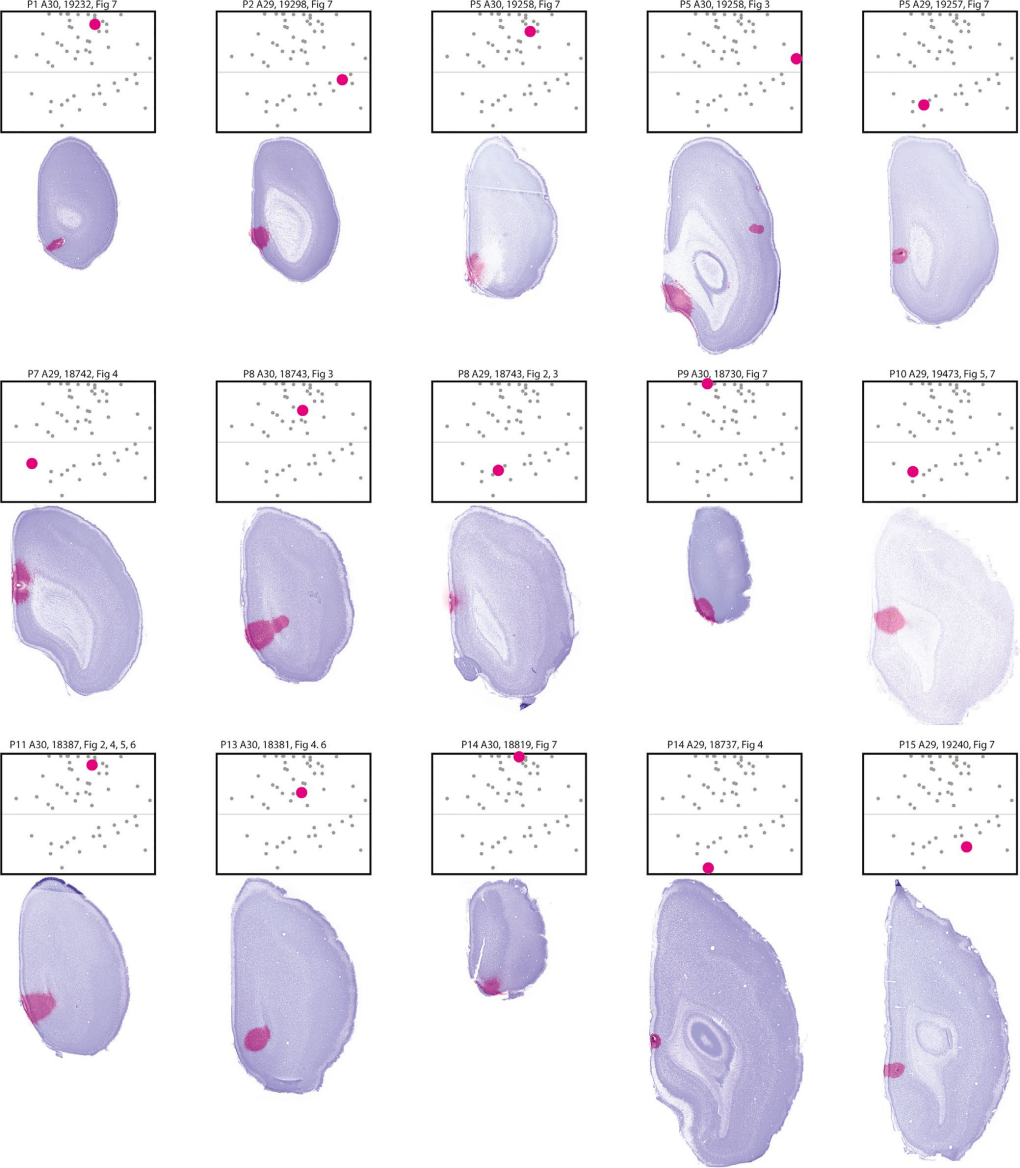
Injections used in figures. Images of the section containing the center of the injection overlaid on the image of the neighboring Nissl‐stained section are shown together with the flatmaps indicating the normalized position of the respective injection (large dot). Smaller dots depict the normalized position of other injections used for analysis. Heading depicts age of the animal at perfusion, area injected, animal identity, and in which figure retrogradely labeled neurons of the injection were displayed. [Colour figure can be viewed at wileyonlinelibrary.com]

As brains of different ages have different sizes, we aimed to normalize the position of the injections in RSC. For this purpose, we took advantage of a reference 3D‐atlas brain (Papp, Leergaard, Calabrese, Johnson, & Bjaalie, 2014, 2015). First, we identified regularly spaced coordinates of the dorsal, ventral, rostral, and caudal borders of, respectively, A29 and A30 in the atlas brain. The lines between the respective coordinates were smoothed using local regression. Next, we calculated the cutting angle of our experimental brains relative to the atlas brain, and generated matching sections of the standard atlas brain with the same cutting angle. To identify the best matching atlas sections, we used landmarks and cytoarchitectonic borders present in the section containing the center of each injection as selection criteria. The atlas coordinate of the center of each injection was obtained and represented as an age‐normalized 3D point‐measure of the injection site within RSC. For illustrative purposes, the injections were plotted within the 3D volume (Figure 1a, ITK‐SNAP, NIH). As caudal RSC is curved both along the dorsoventral and rostrocaudal axis, we flattened RSC and transposed each injection into a 2D plane (Figure 1b). We divided the surface area of A29 and A30 into multiple triangles (for methodological details, see Sugar & Witter, 2016). In short, the coordinates of the corners of these triangles were obtained from the coordinates of the dorsal and ventral borders of A29 and A30. For each injection, we calculated the shortest vector between the injection and the cortical surface within any of the triangles. Thereafter, we calculated the coordinate of the intersection of the vector with the plane within the triangle. This coordinate represented the “transposed” location onto the cortical surface of each injection and effectively flattened the 3D coordinates of the injections into a 2D plane of RSC (Figure 1b). Next, we normalized the dorsoventral and rostrocaudal coordinates of all injections (Figures 1c and 2). The normalized dorsoventral coordinate was defined as the ratio of the distance from the injection to the ventral border of RSC divided by the distance from the dorsal to the ventral border of RSC. The normalized rostrocaudal coordinate was defined as the ratio of the distance from the injection to the rostral pole of RSC divided by the distance from the rostral to the caudal pole of the injection. From this, we derived the normalized rostrocaudal and dorsoventral positions of each injection (Figure 1c).

## 3 RESULTS

### 3.1 Injection sites

To investigate the development of HF and PHR to RSC projection patterns, we injected retrograde tracers in different locations within RSC of differently aged pups. We analyzed 56 injections of retrograde tracers in 38 animals (Figures 1 and 2; Table 1, female *n* = 20; male *n* = 18), providing us with 37 injections in A30 and 19 injections in A29. We regarded each of these injections as independent experiments. Following nine of these injections, we did not observe retrogradely labeled cells in HF‐PHR, although in other areas, such as the thalamus, labeled cells were present. The observation of retrogradely labeled neurons in other brain structures implied that the lack of retrogradely labeled neurons in HF‐PHR probably reflected the absence of HF‐PHR‐to‐RSC projections rather than failure of dye transport. More than half of these experiments were performed in animals younger than P3, however a few older animals also showed similar patterns of retrograde transport (P1, P3, P10 and P14; *n* = 1, P2; *n* = 3, P9; *n* = 2). Most of these injections (*n* = 8) were located in the rostral half of RSC.

**Table 1:** All injections used in analysis

Animal	Age	Sex	Dye	Injection size	Injection site			Labeled neurons observed					
				Vertical and Horizontal	Area	R‐C	Layers	CA1	Sub	PrS/PaS	EC	POR	PER
19232	P1	F	DiI	600 and 400 μm	A30	I	Sup + deep	–	+	+	+	+	–
19470	P1	M	DiI	600 and 670 μm	A30	I	Sup + deep	–	–	–	–	–	–
19297	P2	F	DiI	915 and 630 μm	A29	I	Sup + deep	–	–	–	–	–	–
19297	P2	F	FB	615 and 555 μm	A29	I	Sup + deep	–	–	–	–	–	–
19298	P2	F	DiI	930 and 710 μm	A30	I	Deep	–	–	–	–	–	–
19298	P2	F	FB	920 and 670 μm	A29	C	Sup + deep	–	+	+	+	–	–
19049	P3	F	FB	380 and 450 μm	A29	C	Sup + deep	–	++	+	–	–	–
19299	P3	F	DiI	300 and 270 μm	A29	R	Sup	–	+	+	+	–	–
19299	P3	F	FB	240 and 310 μm	A30	I	Sup + deep	–	–	–	–	–	–
19257	P5	M	DiI	500 and 450 μm	A30	R	Sup + deep	–	+	++	+	+	–
19257	P5	M	FB	600 and 570 μm	A29	R	Sup + deep	–	+++	+	–	–	–
19258	P5	M	DiI	200 and 510 μm	A30	C	Sup + deep	–	++	++	+	+	–
19258	P5	M	FB	900 and 1,100 μm	A30	C	Sup + deep	+	+++	+++	+	+	–
18810	P6	M	FB	400 and 370 μm	A30	I	Sup + deep	–	+++	++	+	–	–
18810	P6	M	DiI	210 and 610 μm	A30	I	Sup + deep	–	+++	++	+	–	–
19259	P6	M	FB	700 and 740 μm	A29	I	Sup + deep	–	+	+	–	–	–
18379	P6	F	FB	920 and 1,000 μm	A30	C	Deep	–	+++	+++	+++	+++	–
18391	P7	M	FB	800 and 1,200 μm	A30	C	Sup + deep	–	+++	+++	+++	+++	–
18742	P7	M	DiI	340 and 400 μm	A29	R	Sup + deep	–	+++	+++	+	–	–
18742	P7	M	FG	300 and 450 μm	A29	R	Sup	+	+++	+++	++	–	–
19260	P7	M	DiI	100 and 510 μm	A30	I	Sup + deep	–	+++	+++	+	–	–
18743	P8	F	DiI	400 and 250 μm	A29	R	Sup	+	+++	++	+	–	–
18743	P8	F	FB	1,400 and 1,200 μm	A30	I	Sup + deep	–	+++	+++	++	+++	–
18943	P8	F	IX 488	300 and 450 μm	A30	I	Sup	+	+++	++	+	+++	–
19127	P8	M	DiI	315 and 250 μm	A29	C	Sup + deep	–	++	+	+	+	–
19127	P8	M	FB	360 and 550 μm	A30	C	Deep	+	+++	+++	+	++	–
19235	P8	M	DiI	1,300 and 580 μm	A30	C	Sup + deep	+	+++	+++	++	++	–
18385	P9	F	DiI	90 and 130 μm	A30	I	Deep	–	–	–	–	–	–
18385	P9	F	FB	1,400 and 1,200 μm	A30	C	Sup + deep	+	+++	+++	+++	+++	–
18730	P9	F	FB	950 and 780 μm	A30	I	Sup + deep	+	++	++	++	++	–
18730	P9	F	DiI	300 and 450 μm	A30	I	Sup	–	++	++	+	+	–
19051	P9	M	DiI	200 and 390 μm	A29	I	Deep	–	+++	+	–	–	–
19236	P9	M	FB	910 and 1,050 μm	A30	I	Sup + deep	+	+++	+++	+++	++	–
19236	P9	M	DiI	270 and 200 μm	A30	I	Deep	–	–	–	–	–	–
18380	P10	F	FB	2,030 and 980 μm	A30	R	Deep	–	+++	+++	+++	+++	+
19237	P10	F	DiI	340 and 390 μm	A30	I	Deep	–	+	+	–	+	–
19237	P10	F	FB	370 and 320 μm	A30	I	Deep	–	–	–	–	–	–
19473	P10	F	FB	520 and 510 μm	A29	R	Deep	+	+++	++	+	–	–
19473	P10	F	DiI	220 and 250 μm	A29	C	Deep	–	+++	+	–	–	–
18387	P11	F	FB	1,300 and 940 μm	A30	I	Sup + deep	+	+++	+++	+++	+++	–
19239	P11	F	DiI	360 and 520 μm	A30	I	Sup + deep	–	+++	+	–	–	–
19455	P11	F	FB	780 and 610 μm	A30	I	Sup + deep	–	+++	+++	–	++	–
19053	P12	M	DiI	780 and 610 μm	A29	R	Sup + deep	+	+++	+	+	–	–
19053	P12	M	FB	130 and 140 μm	A29	I	Sup	–	+	–	–	–	–
19215	P12	M	FB	440 and 560 μm	A30	I	Deep	+	+++	+++	++	++	–
19216	P12	F	FB	310 and 290 μm	A30	I	Sup + deep	+	+	+	–	–	–
18381	P13	F	FB	820 and 870 μm	A30	I	Deep	–	+++	+++	+++	+++	–
19210	P13	M	FB	580 and 660 μm	A30	I	Sup + deep	+	+	+	+	+	–
19210	P13	M	DiI	1,100 and 930 μm	A30	C	Sup + deep	–	+++	+	+	–	–
19217	P13	M	FB	290 and 320 μm	A30	I	Deep	+	+	+	–	+	–
18746	P14	F	FB	630 and 700 μm	A30	R	Sup + deep	–	–	–	–	–	–
18746	P14	F	DiI	300 and 450 μm	A29	C	Sup + deep	+	+++	+	–	–	–
18819	P14	M	DiI	700 and 800 μm	A30	I	Sup + deep	–	+	+	+	++	–
18737	P15	F	FB	400 and 560 μm	A29	R	Sup	+	+++	+++	+++	+	+
19240	P15	M	DiI	340 and 340 μm	A30	C	Sup + deep	+	+	+	+	+	–
19240	P15	M	FB	580 and 650 μm	A29	I	Sup + deep	–	+++	++	+	+	–

List of all injections used in experiments including the animal identity, age at perfusion, sex, dye injected, size of the injection (diameter in the horizontal and vertical plane), area containing the center of the injection (A29 or A30), the rostrocaudal level of the injection (R: rostral, I: intermediate rostrocaudal and C: caudal levels of RSC), and whether injection covered deep and/or superficial layers of RSC. Columns to the right include information of whether we observed labeled cells (+: <5, ++: 5–10 cells in a section, +++: more than 10 cells in a section) in CA1, subiculum (Sub), pre‐ and parasubiculum (Prs/PaS), entorhinal cortex (EC), postrhinal cortex (POR), or perirhinal cortex (PER).

### 3.2 General distribution patterns

In accordance with previous studies in the adult, we observed retrogradely labeled neurons in the anterior nuclei of the thalamus, anterior cingulate cortex, prelimbic cortex, orbitofrontal cortex, parietal and visual cortices, in the claustrum, and the septal complex (van Groen & Wyss, 1990, 1992, 2003; Jones, Groenewegen, & Witter, 2005). However, a detailed assessment of these projections was outside the scope of this paper. Within HF‐PHR, we observed a few retrogradely labeled neurons in CA1 after some of the injections. In SUB, as well as in PrS and PaS, we observed many retrogradely labeled neurons. In PrS and PaS, most labeled neurons were located in layers V–VI. Some of the injections resulted in a moderate number of retrogradely labeled neurons in layers III and V–VI of MEC and in layers II–VI of postrhinal cortex (POR), whereas in only a few cases, we observed a few retrogradely labeled neurons in layers V–VI of perirhinal cortex (PER).

### 3.3 Labeling in CA1

**Figure 3:**
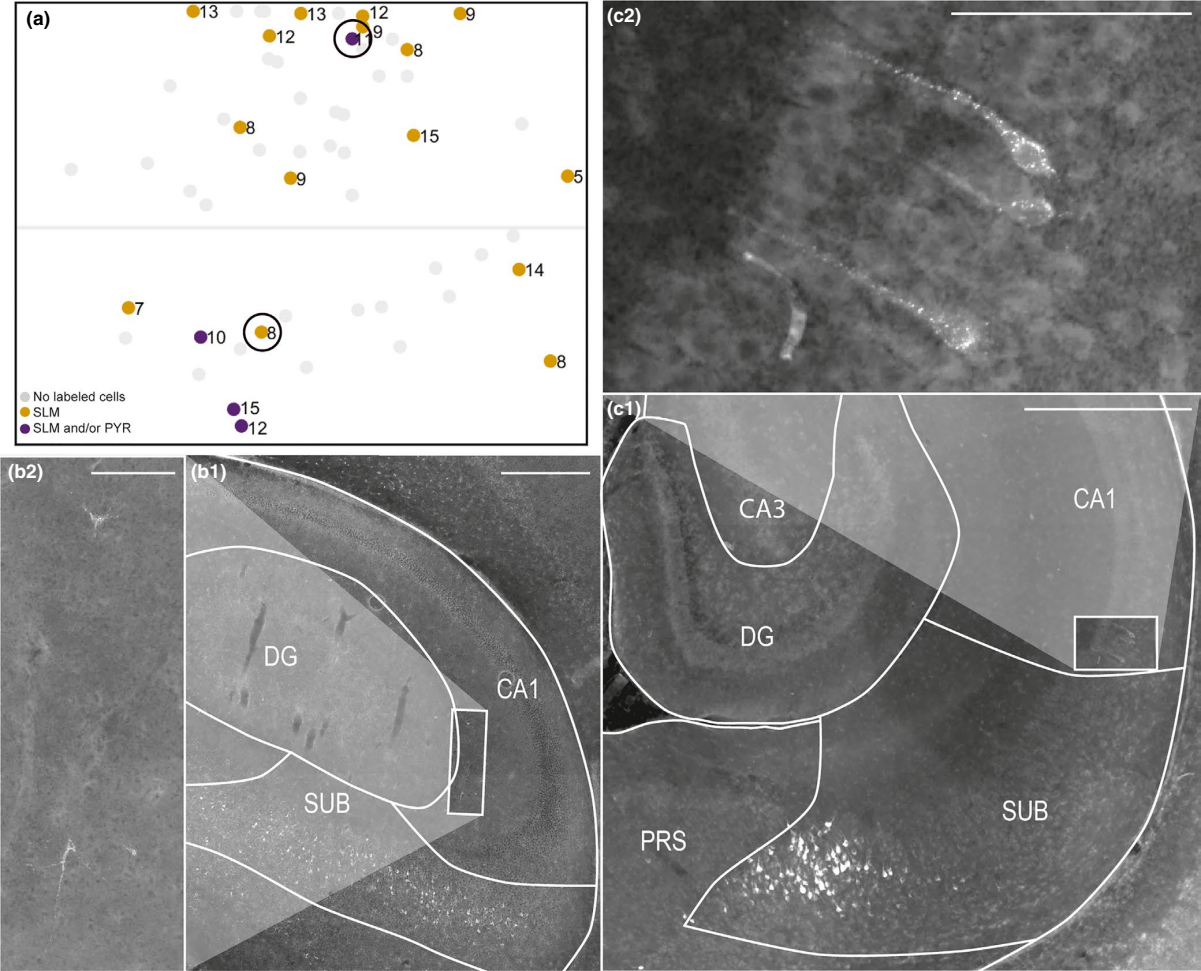
Labeled neurons in CA1. (a) Normalized flatmap of RSC. Dots represent injections resulting in retrogradely labeled neurons in the pyramidal layer and/or the stratum lacunosum moleculare and/or stratum radiatum of CA1. Light gray dots represent injections not resulting in retrogradely labeled neurons in CA1. Black circles indicate examples used in the figure (b) and (c). (b) A representative example of retrogradely labeled neurons in stratum radiatum of CA1 after an injection in A29 (animal 18743 P8, DiI). White square in B1 depicts the area shown in high power in B2. The retrogradely labeled neurons had a non‐pyramidal‐shaped soma and several dendrites leaving the soma in several directions. Scale bar: 500 μm (b1) and 100 μm (b2). (c) A representative example of retrogradely labeled neurons in the pyramidal layer of CA1 after an injection in A30 (animal 18387 P11, FB). White square in c1 depicts the area shown in high power in c2. The retrogradely labeled neurons had a pyramidal‐shaped soma and a thick apical dendrite. Many retrogradely labeled neurons could also be seen in distal SUB. Scale bar: 500 μm (c1) and 100 μm (c2). White lines in B and C depict borders of hippocampal and parahippocampal subregions. [Colour figure can be viewed at wileyonlinelibrary.com]

Nineteen of the injections (34%) resulted in a few retrogradely labeled neurons (typically 1–5 neurons in each section) in stratum radiatum and/or in stratum lacunosum moleculare of the most anterior part of dorsal CA1 (Figure 3a,b). The retrogradely labeled neurons generally had a non‐pyramidal‐shaped soma with several dendrites leaving the soma in several directions. In four of these cases (7% of the injections), we additionally observed single retrogradely labeled neurons (typically 1–3 neurons within a section) in the pyramidal layer of dorsal and/or intermediate dorsoventral CA1 (Figure 3a,c). The neurons generally had a pyramidal‐shaped soma with a thick clearly visible apical dendrite. Such neurons were observed after three injections in A29 located close to the corpus callosum, and after one injection very dorsally in A30.

### 3.4 Labeling in SUB

**Figure 4:**
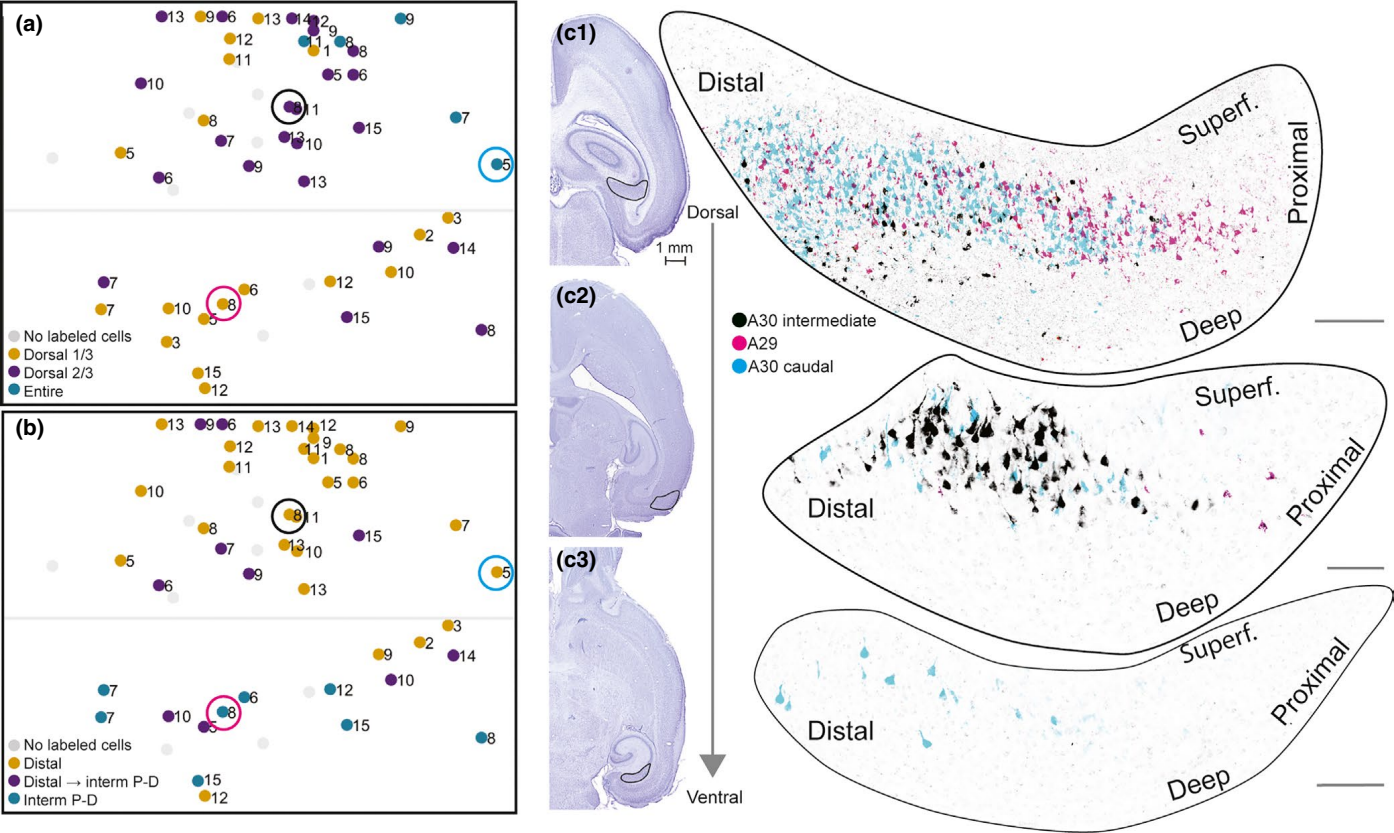
Labeled neurons in SUB. (a) Normalized flatmap of RSC illustrating the dorsoventral organization of the origin in SUB of projections to RSC. Injections resulting in retrogradely labeled neurons in the dorsal one‐third of SUB (yellow dots), the dorsal two‐thirds of SUB (purple dots), and the entire SUB (green dots). Gray dots depict injections not resulting in labeled neurons in SUB. Injections in the caudal A30 resulted in retrogradely labeled neurons in the entire SUB, whereas progressively more rostral and ventral injections resulted in retrogradely labeled neurons in more dorsal parts of SUB. (b) Normalized flatmap of RSC illustrating the proximodistal organization of the origin in SUB of projections to RSC. Injections were color‐coded based on the distribution of the retrogradely labeled neurons: in the distal one‐third of SUB (yellow dots), in distal SUB dorsally and progressively more proximal SUB at more ventral levels (purple dots), and in intermediate proximodistal SUB (green dots). Gray dots depict injections not resulting in labeled neurons within SUB. Injections in dorsal RSC resulted in retrogradely labeled neurons in distal SUB and progressively more ventral injections resulted in retrogradely labeled neurons in more proximal parts of SUB. (c) Representative examples of the distribution of retrogradely labeled neurons shown in three horizontal sections (c1–c3), taken at different dorsoventral levels after three injections. The distributions of the three cases were merged into one representation in order to illustrate the complex topography along the dorsoventral and proximodistal axes of the subiculum. Retrogradely labeled neurons after a caudal injection in A30 (cyan, 19258 P5, FB), an intermediate rostrocaudal A30 injection (black, 18743 P8, FB), and a ventral and more rostral A29 injection (magenta, 18743 P8, DiI). Circles in (a) and (b) depict the location of each injection in RSC. High‐power images (right) of dorsal (top) and more ventral levels of SUB (bottom) are shown. Black contours in adjacent Nissl‐stained sections (left) depict location of high‐power images. Injections in A30 resulted in retrogradely labeled neurons in distal SUB (cyan and black), whereas injections in A29 resulted in retrogradely labeled neurons in more proximal SUB (magenta). Injections in caudal A30 (cyan) resulted in retrogradely labeled neurons along most of the dorsoventral level of distal SUB, whereas rostral injections resulted in labeled neurons in only the dorsal SUB (magenta and black). Scale bar: 100 μm. [Colour figure can be viewed at wileyonlinelibrary.com]

All injections resulting in retrogradely labeled neurons within HF‐PHR, displayed retrogradely labeled neurons in the pyramidal layer of SUB. Additionally, SUB was the HF‐PHR subregion that in every such experiment contained the highest number of retrogradely labeled neurons. We observed a marked topographical distribution of the labeled neurons in SUB, depending on the location of the injection. First, caudal injections within A30 resulted in retrogradely labeled neurons in the entire dorsoventral SUB, whereas progressively more rostral and ventral injections in A30 and A29 resulted in retrogradely labeled neurons, preferentially in more dorsal parts of SUB (Figure 4a). As illustrated, an injection in caudal A30 (cyan) resulted in retrogradely labeled neurons along the entire dorsoventral extent of SUB (Figure 4c1–3). In contrast an intermediately positioned injection in A30 (black), like most of the caudal injections in A29, resulted in retrogradely labeled neurons in the dorsal two‐thirds of SUB (Figure 4c1–2). An injection in the rostral half of A29, like most of the rostral injections in A30, resulted in retrogradely labeled neurons in only the dorsal one‐third of SUB (Figure 4a,c1, magenta).

The distribution of retrogradely labeled neurons also showed a clear topography along the proximodistal axis of SUB (Figure 4b,c). Injections in A30 resulted in retrogradely labeled cells in the distal extreme of SUB. Injections in A29 resulted in retrogradely labeled neurons in intermediate proximodistal SUB. We never found retrogradely labeled neurons in the proximal extreme of SUB. A few injections located in A29 and adjacent A30 resulted in retrogradely labeled cells in both distal and intermediate proximodistal SUB. The retrogradely labeled cells in distal SUB were always positioned at dorsal levels of SUB, whereas the retrogradely labeled cells in intermediate proximodistal parts were located in more ventral levels of SUB. This mixed pattern suggests that A29 and the directly adjacent parts of A30 receive input from both distal parts of dorsal SUB and intermediate proximodistal parts of more ventral SUB. In general, both young and older rats displayed similarly organized topographical distribution patterns, indicating the absence of clear effects of age, both with respect to the dorsoventral and the proximodistal location of retrogradely labeled cells in SUB.

### 3.5 Labeling in PrS and PaS

**Figure 5:**
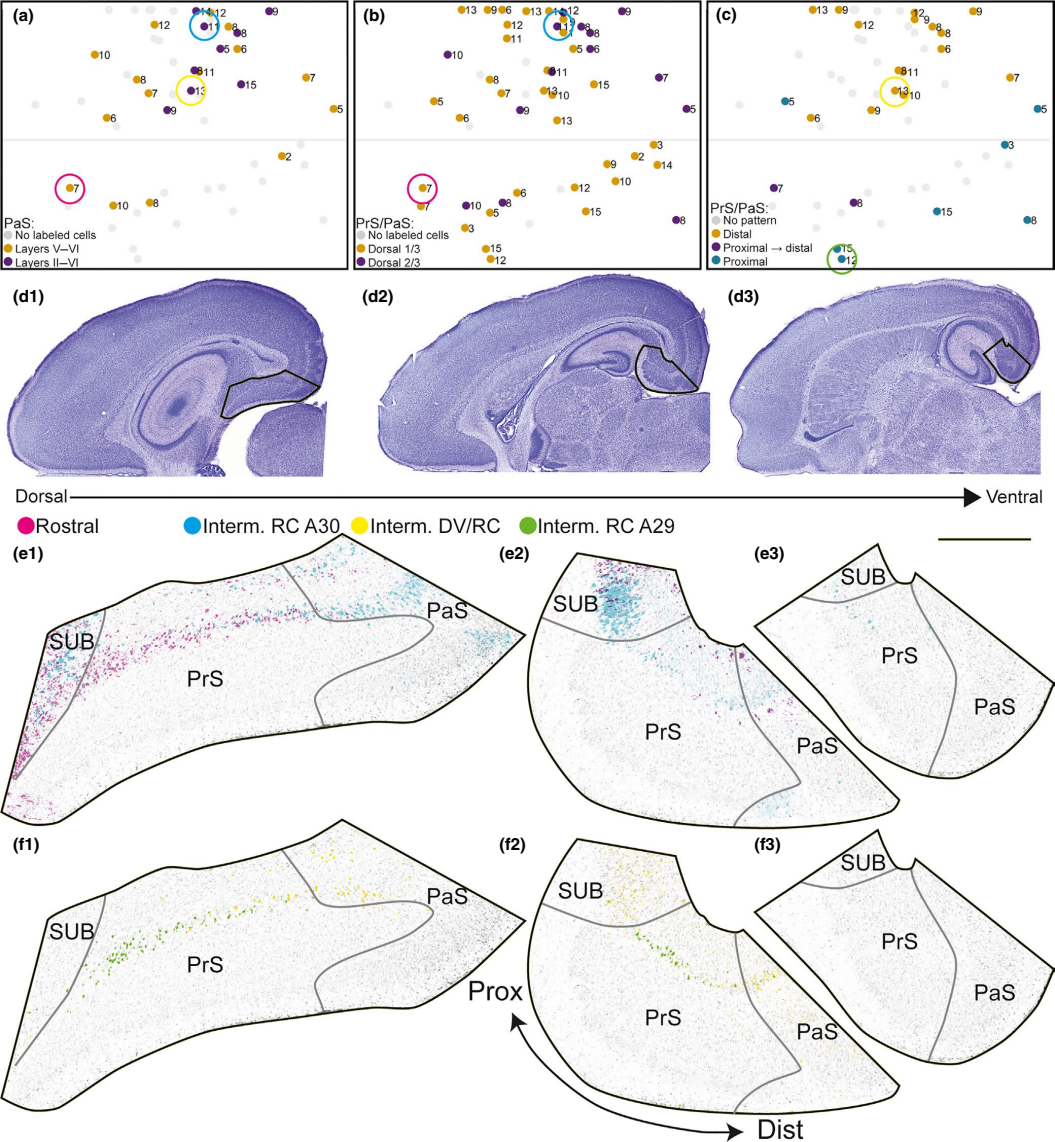
Labeled neurons in PrS and PaS. (a) Normalized flatmap of RSC illustrating the superficial‐to‐deep organization of the origin of RSC projections in PaS. Injections were color‐coded based on the distribution of the retrogradely labeled neurons: in only layer V of PaS (yellow dots), or layer V and layers II–III of PaS (purple dots). Gray dots depict injections with no labeled neurons in PaS. Injections resulting in retrogradely labeled neurons in layers II–III of PaS (purple dots) are located in caudal A30. In PrS, labeled neurons were only seen in deep layer V and VI. No retrogradely labeled neurons were observed in superficial layers of PrS. Color‐coded circles in A–C depict the location of injections in RSC, shown in figures (e,f). (b) Normalized flatmap of RSC illustrating the dorsoventral organization of the origin of RSC projections in PrS and PaS. Injections resulting in retrogradely labeled neurons in the dorsal one‐third (yellow dots) and dorsal two‐thirds of layer V of PrS and PaS (purple dots) are indicated. Gray dots depict injections with no labeled neurons in PrS or PaS. (c) Normalized flatmap of RSC illustrating the proximodistal organization of the origin of RSC projections in PrS and PaS. Injections resulting in retrogradely labeled neurons in only distal parts of layer V of PrS and the transverse extent of PaS (yellow dots), proximal PrS at dorsal levels and progressively more distal parts of layer V of PrS and PaS at more ventral levels (purple dots), and in proximal parts of layer V of PrS (green dots) are indicated. Gray dots depict injections that did not display a clear patterned distribution of retrogradely labeled cells in PrS/PaS or did not contain retrogradely labeled neurons. Injections resulting in retrogradely labeled neurons in distal PrS and the whole PaS were located in A30, whereas A29 contained the injections resulting in retrogradely labeled neurons in proximal PrS. (d) Nissl stains of sections from animal 18387 (P11). Sections were used as background in E and F. Black contours depict location of high‐power images in (e) and (f). (e) Representative examples of the distribution of retrogradely labeled neurons shown in three horizontal sections (e1–e3), taken at different dorsoventral levels after two injections in different animals of different ages. The injections were located in rostral A29 (magenta; 18742 P7) and centered in A30 (cyan; 18387 P11). Color‐coded circles in (a) and (b) depict the location of each injection in RSC. High‐power images of dorsal (left) and more ventral levels of SUB, PrS, and PaS (right) are shown. The injection centered in A30 (cyan) resulted in retrogradely labeled neurons in SUB, LV/VI of PrS and LII/III/V/VI of PaS. Retrogradely labeled neurons were present in dorsal levels (e1–2) of PrS and PaS and in ventral parts of PrS (e3). The very rostral injection in A29 (magenta) resulted in retrogradely labeled cells in SUB and in layer V/VI of PrS and PaS. Retrogradely labeled neurons were located in the dorsal part of PrS (e1–2). Scale bar: 500 μm. (f) Representative examples of the distribution of retrogradely labeled neurons. Sections are organized as in E. The injections were centered in A30 (yellow, 18381 P13) and centered in ventral A29 (green 18737 P15). The injection in A30 resulted in retrogradely labeled cells in layer V/VI of distal PrS and PaS, whereas the injection in A29 resulted in retrogradely labeled neurons in layer V/VI of proximal PrS. Both cases displayed retrogradely labeled neurons in SUB and neither displayed retrogradely labeled neurons in superficial layers of PaS. [Colour figure can be viewed at wileyonlinelibrary.com]

All injections resulting in retrogradely labeled neurons within HF‐PHR resulted in retrogradely labeled neurons in PrS and PaS. We observed retrogradely labeled neurons in superficial parts of layer V of PrS and PaS, directly deep to the lamina dissecans. The labeled neurons were positioned in a “band” of cells often in direct continuation with the retrogradely labeled neurons in distal SUB. Some injections also labeled neurons in deep parts of layer VI. In PrS, we rarely observed retrogradely labeled cell in layers II–III, whereas in PaS, we observed retrogradely labeled cells in superficial layers after nine injections (16% of all injections). All of these latter injections were located in the caudal half of A30 (Figure 5a).

The labeled neurons in PrS and PaS displayed a distinct topography dependent on the location of the injection within RSC (Figure 5b–f). Injections located caudally in A30 tended to result in retrogradely labeled cells more ventrally in PrS and PaS, covering the dorsal two‐thirds of PrS and PaS. Injections in more rostral parts of A30 and the complete extend of A29 preferentially resulted in retrogradely labeled cells in only the most dorsal one‐third of PrS. As illustrated, an injection centered in A30 (cyan, Figure 5b,e), like most of the caudal injections in A30 resulted in retrogradely labeled neurons in the dorsal two‐thirds of PrS. In contrast, an injection in the rostral extreme of A30 resulted in retrogradely labeled neurons in the dorsal extreme of PrS (magenta, Figure 5b,e).

We further observed in a minority of the experiments that retrogradely labeled neurons showed differential distributions along the proximodistal axis of PrS and PaS. In the cases which displayed such patterns, injections in A30 resulted in labeled neurons in distal PrS and the whole PaS (yellow, Figure 5c,f). In contrast, after injections in A29, retrogradely labeled neurons were confined to proximal parts of PrS (green, Figure 5c,f). In these cases, we did not observe retrogradely labeled cells in distal PrS and PaS. In two cases, we observed retrogradely labeled cells in both proximal PrS at dorsal levels and in more distal PrS and throughout the transverse extent of PaS at more ventral levels. The centers of these injections were located in rostral A30 and in the rostrocaudal middle of A29. A mixed topographical organization suggests that there are areas in RSC receiving inputs from both proximal and distal PrS. To our knowledge such a pattern has not been observed in the adult. However, the limited number of observed cases makes it difficult to draw clear conclusions. The dorsoventral and proximodistal organization of retrogradely labeled cells were present in both the youngest and older animals so we conclude that this topographical organization is independent of age.

### 3.6 Labeling in EC

**Figure 6:**
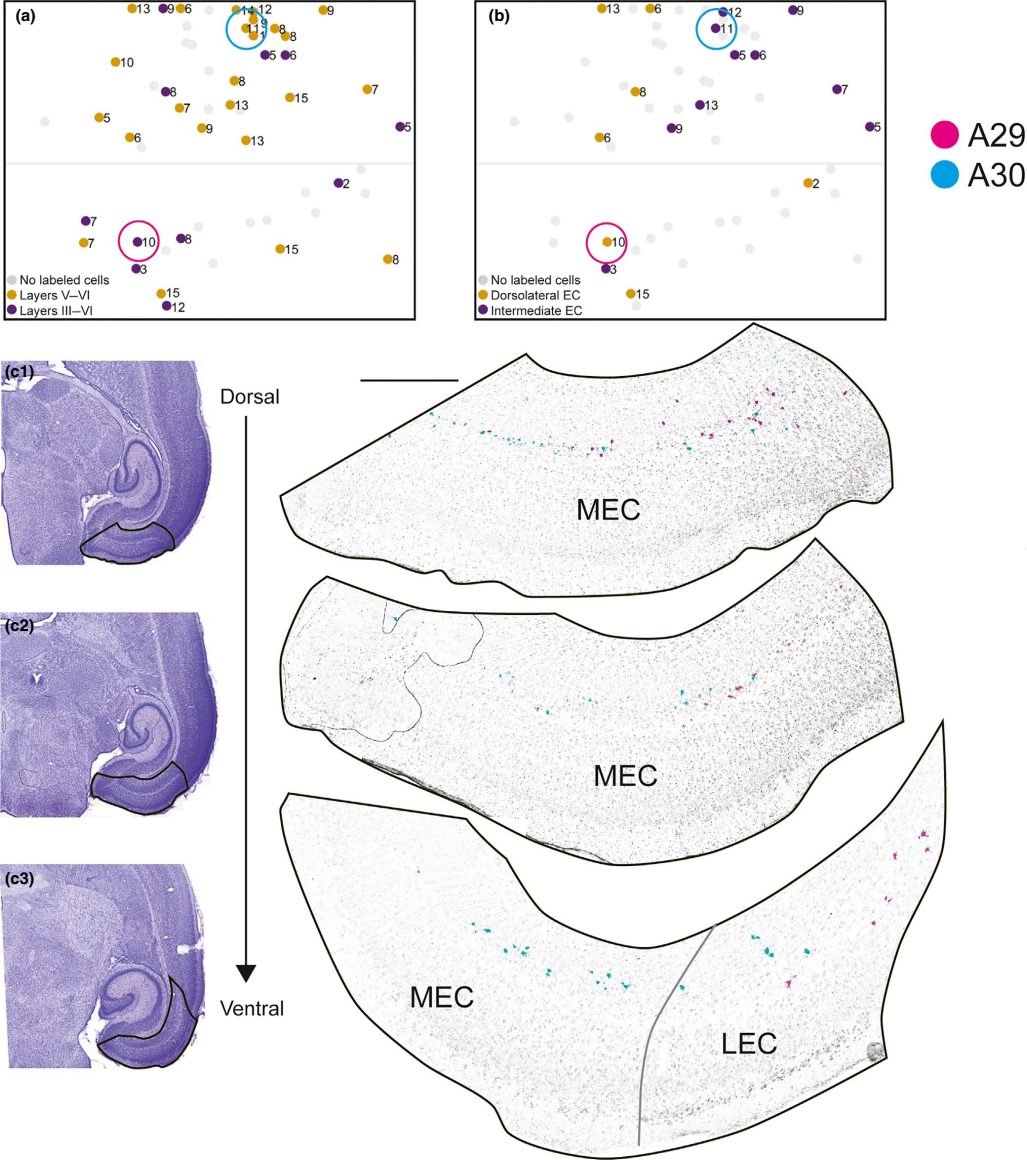
Labeled neurons in entorhinal cortex. (a) Normalized flatmap of RSC illustrating the superficial‐to‐deep organization of origin of RSC projections in EC. Injections were color‐coded based on the distribution of the retrogradely labeled neurons: injections resulting in retrogradely labeled neurons in layers V and VI of entorhinal cortex (orange dots), and both layers V–VI and layer III of entorhinal cortex (purple dots). Gray dots depict injections resulting in no retrogradely labeled neurons in entorhinal cortex. (b) Normalized flatmap of RSC illustrating the mediolateral organization of origin in EC. Injections were color‐coded based on the distribution of the retrogradely labeled neurons: injections resulting in retrogradely labeled neurons in dorsolateral EC (yellow dots) and intermediate dorsolateral‐ventromedial EC (purple dots). Gray dots depict injections resulting in no clear dorsolateral‐ventromedial organization of retrogradely labeled neurons in EC or injections with no retrogradely labeled neurons observed in EC. Injections resulting in retrogradely labeled cells in intermediate dorsolateral‐ventromedial EC were located mainly in caudal A30, whereas caudal A30 received projections from dorsolateral EC. Color‐coded circles in A, B depict the location of injections in RSC, shown in figure (c). (c) Representative examples of the distribution of retrogradely labeled neurons shown in three horizontal sections (c1–c3), taken at different dorsoventral levels after two FB injections in RSC. The distributions of the two cases were merged into one representation to illustrate the complex topography along the dorsomedial to ventrolateral axes of entorhinal cortex. The injections were located in A29 (magenta, 19473 P10) and A30 (cyan, 18387 P11). Circles in A and B depict location of each injection in RSC. High‐power images (right) of dorsal (top) and more ventral levels of SUB (bottom) are shown. Black contours in adjacent Nissl‐stained sections (left) depict location of high‐power images. The injection in A30 resulted in retrogradely labeled cells in intermediate dorsolateral‐ventromedial EC (cyan), whereas the injection in A29 result in retrogradely labeled cells in the dorsolateral EC. Scale bar: 500 μm. [Colour figure can be viewed at wileyonlinelibrary.com]

In 35 of the injections (63%), we observed retrogradely labeled neurons in MEC, whereas labeled neurons in lateral entorhinal cortex (LEC) were very sparse. The number of retrogradely labeled cells in MEC was lower than in SUB, PrS, and PaS. Similar to that seen in PrS and PaS, the retrogradely labeled MEC and LEC neurons were mainly located in superficial parts of layer V with occasional labeling of neurons in layer VI directly adjacent to the angular bundle. In addition, we observed retrogradely labeled neurons in layer III of both MEC and LEC in 11 of the 35 injections (20% of all injections, Figure 6a). In these cases, we typically observed 1–5 retrogradely labeled neurons in layer III in each section.

In all cases showing retrogradely labeled neurons in MEC, labeled neurons in dorsal parts of MEC were more numerous compared with ventral parts of MEC. In addition, the distribution of retrogradely labeled neurons at ventral levels of MEC points to a topographical organization, where the mediolateral location of the neurons relates to the caudorostral position of the injection in RSC. In case of injections located in rostral A30 or A29, we observed retrogradely labeled neurons in lateral MEC (Figure 6b–c; magenta). At more ventral levels, some labeled neurons were also located in the part of LEC directly adjacent to MEC (Figure 6c3). Following injections in caudal RSC, in the present material only involving A30, we observed retrogradely labeled neurons in more medial parts of MEC (Figure 6c; cyan).

### 3.7 Labeling in POR

**Figure 7:**
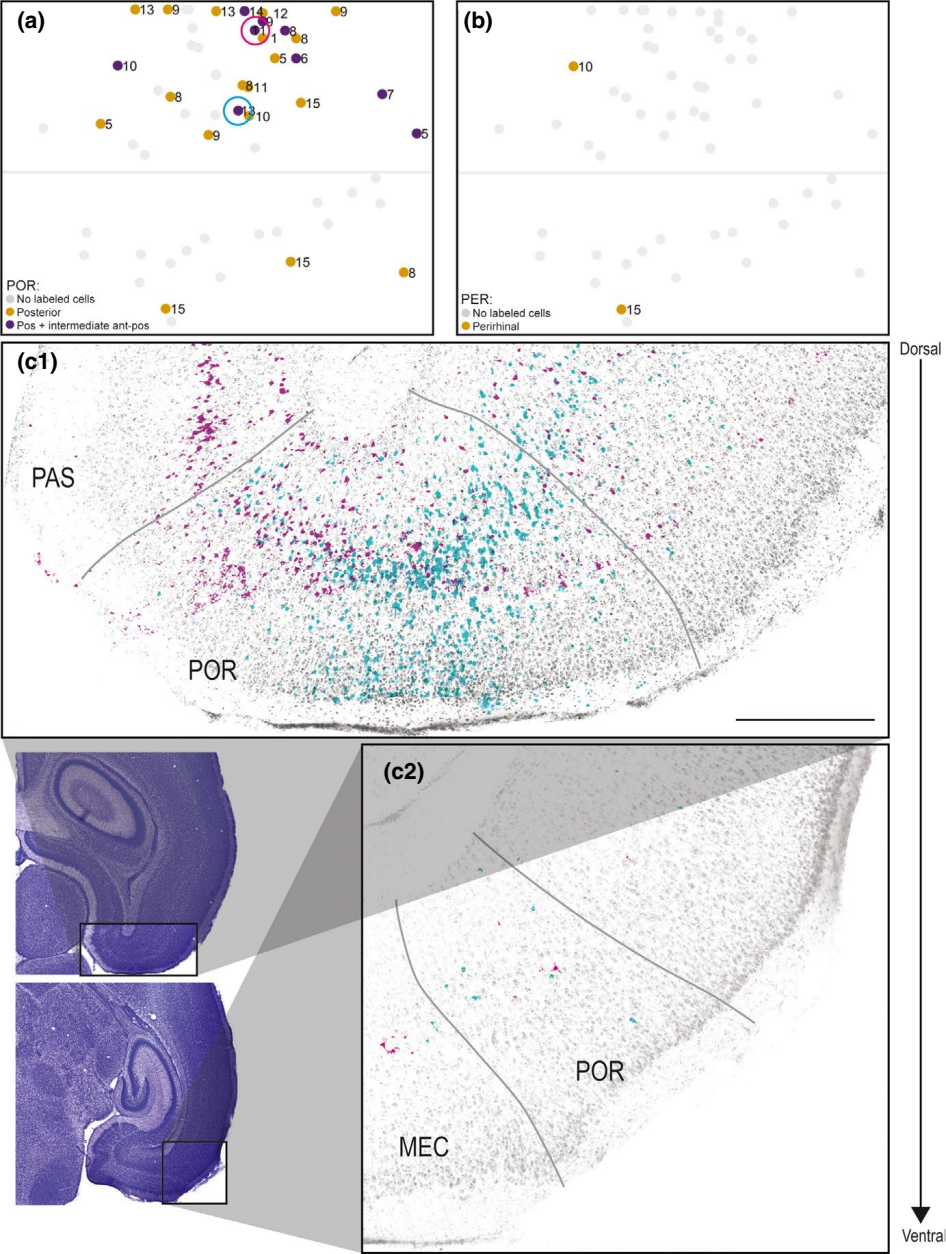
Labeled neurons in POR and PER. (a) Normalized flatmap of RSC illustrating the anteroposterior organization of origin of RSC projections in POR. Indicated are injections resulting in retrogradely labeled neurons in posterior POR (yellow dots) and intermediate anterioposterior POR (purple dots). Gray dots depict injections resulting in no retrogradely labeled neurons in POR. Most of the injections resulting in retrogradely labeled cells in POR were located in A30. Color‐coded circles in A depict the location of injections in RSC, shown in figure (c). (b) Normalized flatmap of RSC illustrating the origin in PER of projections to RSC. Indicated are injections resulting in retrogradely labeled neurons in PER (yellow dots). Gray dots depict injections resulting in no retrogradely labeled neurons in PER. (c) Representative examples of the distribution of retrogradely labeled neurons shown in two horizontal sections, taken at different dorsoventral levels after two FB injections in A30 (cyan, 18381 P13 and magenta, 18387 P11). Representative examples of retrogradely labeled neurons were overlaid on a Nissl section from two posterior to anterior levels of POR (c1 and c2). Circles in A depict the locations of the two injections in RSC. Black contours in adjacent Nissl‐stained sections depict location of high‐power images. Both of the injections resulted in retrogradely labeled cells in deep and superficial layers of POR. In both examples, most of the retrogradely labeled neurons were located in posterior POR (c1), whereas at more anterior levels fewer labeled neurons were visible (c2). Scale bar: 500 μm. [Colour figure can be viewed at wileyonlinelibrary.com]

In 27 of the injections (48%), we observed retrogradely labeled neurons in POR. Of these injections, 24 were located in A30, and only three were located in caudal A29 (Figure 7a). The retrogradely labeled neurons in POR were distributed across layers II–VI, and we observed retrogradely labeled neurons in both the posterior extreme of POR (*n* = 18, Figure 7c1) and in intermediate anteroposterior POR (*n* = 9, Figure 7c2). The injections resulting in retrogradely labeled cells in more intermediate anteroposterior parts of POR were mainly located in the caudal half of A30, whereas the injections resulting in retrogradely labeled neurons in only the posterior POR were located in both rostral and caudal A30. We never observed retrogradely labeled neurons in anterior parts of POR.

### 3.8 Labeling in PER

In only two cases (4%), we observed retrogradely labeled cells in PER (Figure 7b). Typically, we observed 1–5 retrogradely labeled neurons in the most posterior part of PER directly adjacent to POR. These injections, which involved both A29 and A30, were from animals older than P10.

### 3.9 Temporal development of HF‐PHR projections to RSC

**Figure 8:**
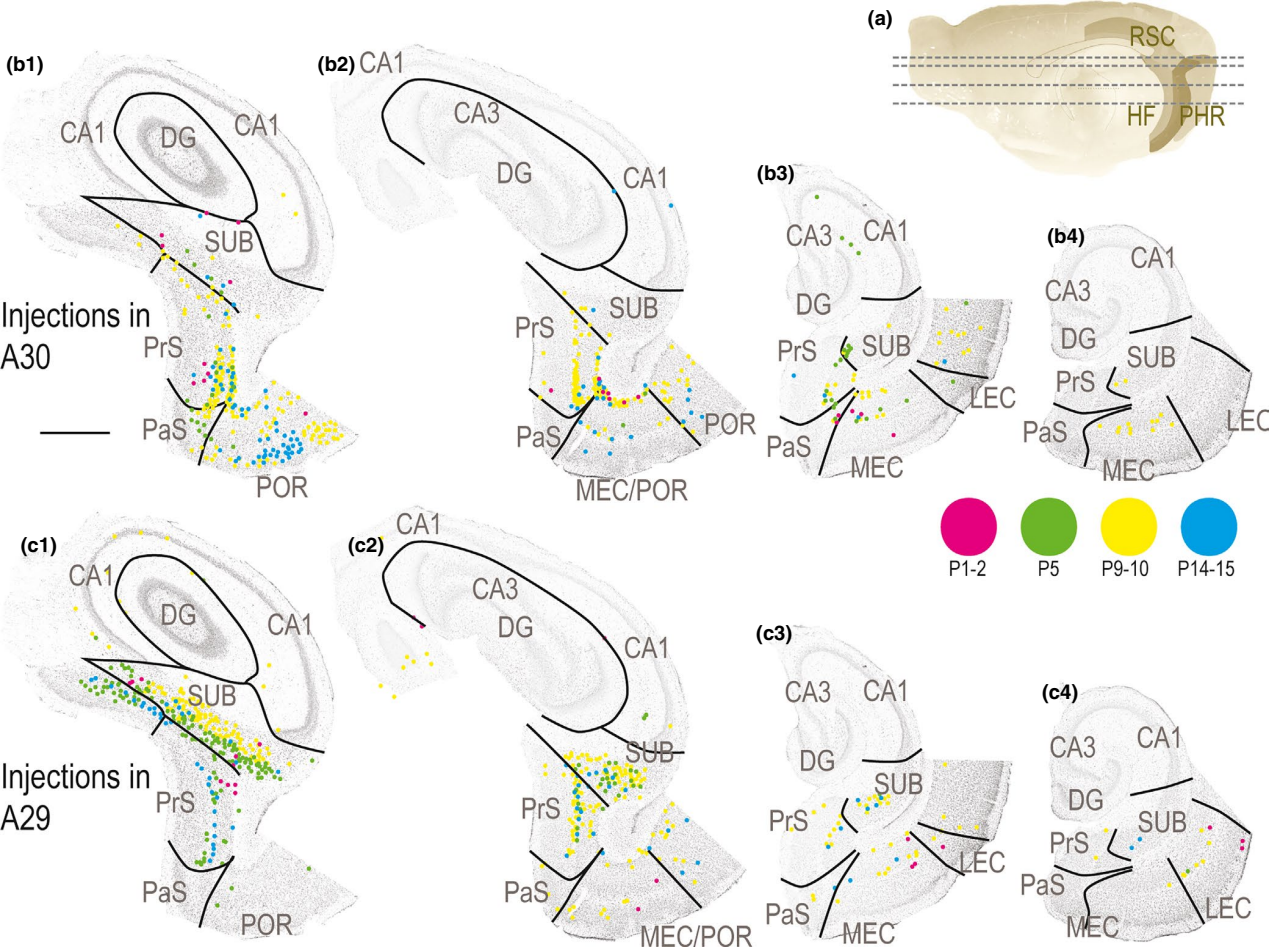
Development of HF‐PHR projections to RSC. (a) Midsagittal view of the rat right hemisphere. Gray lines depict dorsoventral levels of sections in (b) and (c). (b) Retrogradely labeled cells in HF‐PHR after injections in A30. Each dot represents one retrogradely labeled cell and is plotted on Nissl‐stained sections from four dorsal (left) to ventral levels of HF‐PHR (right). Magenta dots represent labeled neurons in a P1 animal (19232 DiI), green dots in a P5 animal (19258 DiI), yellow dots in a P9 animal (18730 FB), and cyan dots in case of a P14 animal (18819 DiI). At all ages, the retrogradely labeled neurons were mainly located in distal SUB, distal PrS, and medial MEC. At the youngest ages (magenta), only a few labeled neurons were seen, whereas in the older ages, a higher number of labeled cells was present. Scale bar: 1,000 μm. (c) Retrogradely labeled cells in HF‐PHR after injections in A29. Panels are organized similar to B. Magenta dots represent labeled neurons in a P2 animal (19298 FB), green dots in a P5 animal (19257 FB), yellow dots in a P10 animal (19473 FB), and cyan dots in case of a P15 animal (19240 FB). At all ages, the retrogradely labeled neurons were mainly located in more proximal parts of SUB compared to after A30 injections, proximal PrS and lateral MEC. At the youngest ages (magenta), only a few labeled neurons were seen, whereas in the older ages, a higher number of labeled cells were present. [Colour figure can be viewed at wileyonlinelibrary.com]

Next, we aimed to study the temporal development of HF‐PHR projections to RSC. We did not observe retrogradely labeled neurons in any of the HF‐PHR subfields following injections in rostral RSC in animals aged P1–3. On the other hand, after injections in the caudal RSC at the same ages, we observed several retrogradely labeled neurons in HF‐PHR. After these injections, most neurons were observed in SUB and PrS, although a few neurons were present in CA1 and MEC and LEC (Figure 8). This suggests that projections to caudal RSC are present already at birth, whereas projections to more rostral parts of RSC started to develop in the second half of the first postnatal week. The labeled cells in SUB, PrS, and MEC were topographically organized similar to the older cases previously described (for instance compare magenta dots in Figure 8b vs. c). Even though we did not quantify the number of retrogradely labeled cells in each experiment, it was obvious that injections in very young animals resulted in fewer retrogradely labeled neurons than injections in older animals. In P1–2 animals, we generally observed up to 10 retrogradely labeled neurons in each section, whereas in older animals this number was substantially higher (Figure 8). This suggests that the overall density of HF‐PHR projections to RSC increases gradually during the postnatal period. Although we observed lower number of retrogradely labeled cells after injections in young animals, it was clear that the distributions of retrogradely labeled neurons after injections in animals aged P1–2 were similar to those resulting from similar injections in older animals (Figure 8). This observation suggests that the first axons arriving in RSC already have a mature topographic organization.

### 3.10 Topographical organization of developing projections

**Figure 9:**
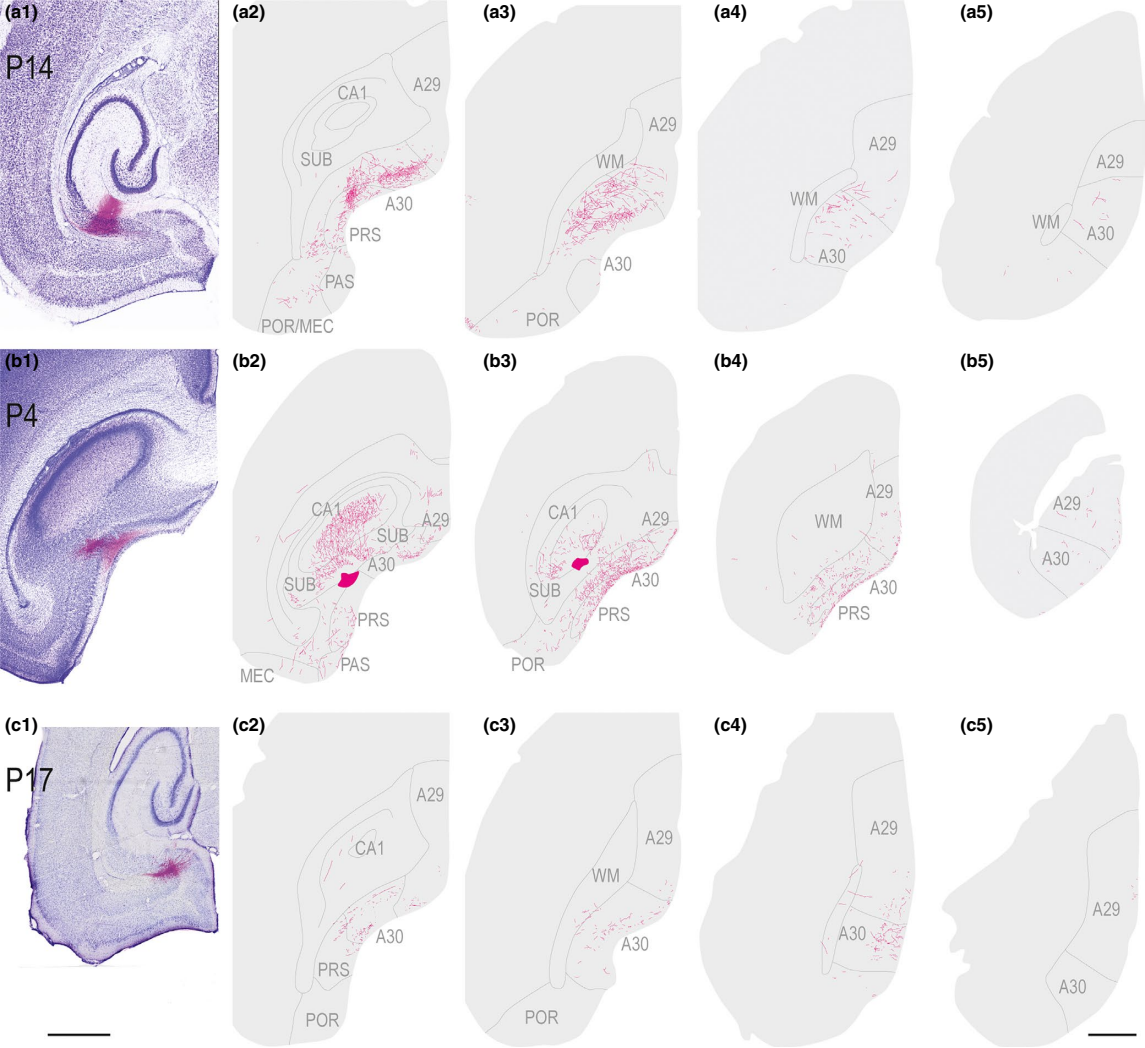
Anterograde injections in subiculum and presubiculum. Left (1): Images of horizontal Nissl‐stained sections overlaid with an adjacent section containing the center of the fluorescent tracer injection. Scale bar; 1,000 μm. Right (2–4): The projections represented in a dorsoventral series of drawings of horizontal sections through RSC. Scale bar: 1,000 μm. (a) A BDA injection in a P14 animal located in distal half of SUB at intermediate dorsoventral levels of SUB. Labeled fibers were present mainly in layer IV and V–VI in A30 and caudal A29. (b) A BDA injection in a P4 animal located at the border between dorsal PrS and dorsal SUB. Labeled fibers were present mainly in layer I and IV of caudal and rostral A30 and A29. (c) A BDA injection in a P17 animal located in deep layers of PrS at intermediate dorsoventral levels. Labeled fibers were present in layer II and V of RSC. [Colour figure can be viewed at wileyonlinelibrary.com]

A subsequent series of anterograde tracing experiments addressed the question whether the developmentally stable topographical distribution of originating neurons in hippocampus and parahippocampus was reflected by a preserved topographical organization of their axons in RSC. We analyzed results from anterograde injections in three of the main input areas, SUB, MEC, and PrS. After anterograde injections in the distal half of SUB at P14 (Figure 9a), labeled axons were present mainly in RSC area 30, in layer I, and layer IV, although a few labeled fibers where present in layer V and VI. A similar projecting pattern was observed in all ages assessed. As an example, an injection at the border between dorsal SUB and dorsal PrS in a P4 animal confirmed that axons were present in RSC already during the first postnatal week (Figure 9b). In this case, labeled fibers were mainly present in caudal A30 and A29, whereas a lower number of fibers were present in rostral parts of A29 and A30. Similarly placed distal injections, but involving more ventral levels of SUB, showed similar results. We also analyzed six injections in proximal SUB (age of injected animal ranging from P1 to P17). We did not identify any labeled axons in RSC in these latter experiments. After an injection in deep layers of the intermediate dorsoventral PrS, labeled fibers were located mainly in layer IV of RSC although layer I contained some labeled fibers as well (Figure 9c). These fibers were mainly located in caudal A30, whereas a few fibers were located in caudodorsal A29.

**Figure 10:**
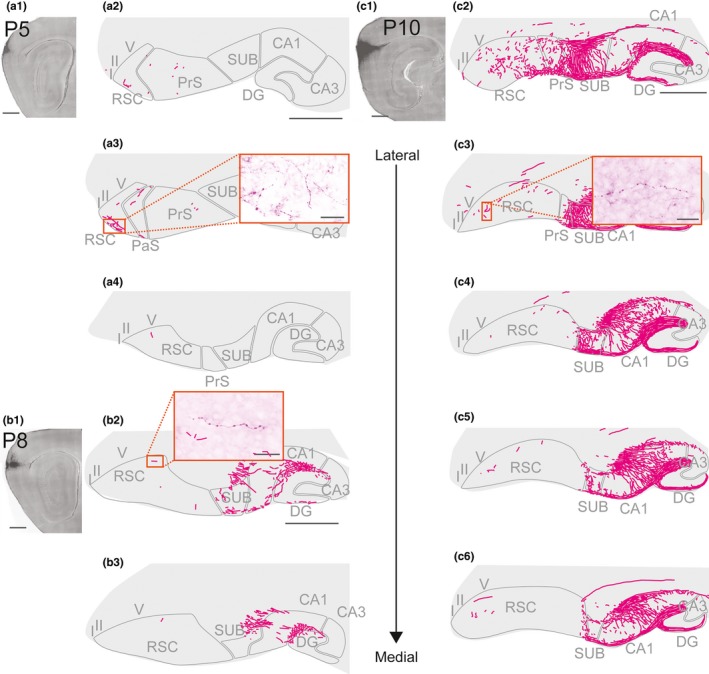
Anterograde injections in MEC. Three representative experiments with anterograde injections in dorsal MEC. Images of injections (left hand upper panels) were taken with a fluorescent microscope. The projections after the injection were traced and represented in a lateral (top) to medial (bottom) series of drawings of sagittal sections through RSC, hippocampus, and parahippocampus. (a) A BDA injection in a P5 animal located in dorsal MEC. A few labeled fibers were present in layer I of RSC. (a1) and (a2); Scale bar: 1,000 μm. A5; Scale bar: 50 μm. (b) A BDA injection in a P8 animal located in dorsal MEC. A few labeled fibers were present in layer I and V of RSC. A dense projection is seen in HC. (b1) and (b2); Scale bar: 1,000. B4; Scale bar: 25 μm. (c) A BDA injection in a P10 animal located in dorsal MEC. Labeled fibers were present in layer I and V of RSC. A dense projection is seen in HC. (c1) and (c2); Scale bar: 1,000 μm. C7; Scale bar: 50 μm. [Colour figure can be viewed at wileyonlinelibrary.com]

We also assessed 11 anterograde injections located in superficial and/or deep layers of dorsal‐to‐intermediate MEC (age of injected animals ranging from P3 to P15). Five of these injections (age = P5, P8, P10, and P12) were localized in dorsolateral MEC and resulted in axon labeling in caudal RSC (Figure 10). Labeled fibers were mainly found in all layers of A29 close to the border between RSC and PrS/PaS/SUB. A few fibers were also seen in deep layers of the caudal pole of RSC near the border to visual cortex. In none of the animals did we observe labeled fibers in rostral parts of rostral A29 or rostral A30. The remaining six injections were localized in the intermediate dorsoventral MEC. In these animals, we did not observe any labeled fibers in RSC. A few fibers in the cingular bundle were observed in two of these cases (not illustrated). The failure to observe labeled axons in RSC was not caused by failure of anterograde transport of the tracer as we observed densely labeled axonal plexuses in DG, CA1‐3, and SUB. We therefore conclude that the axonal distribution of hippocampal and parahippocampal projections to RSC displays a similar topographical organization in all ages present in our material.

## 4 DISCUSSION

**Figure 11:**
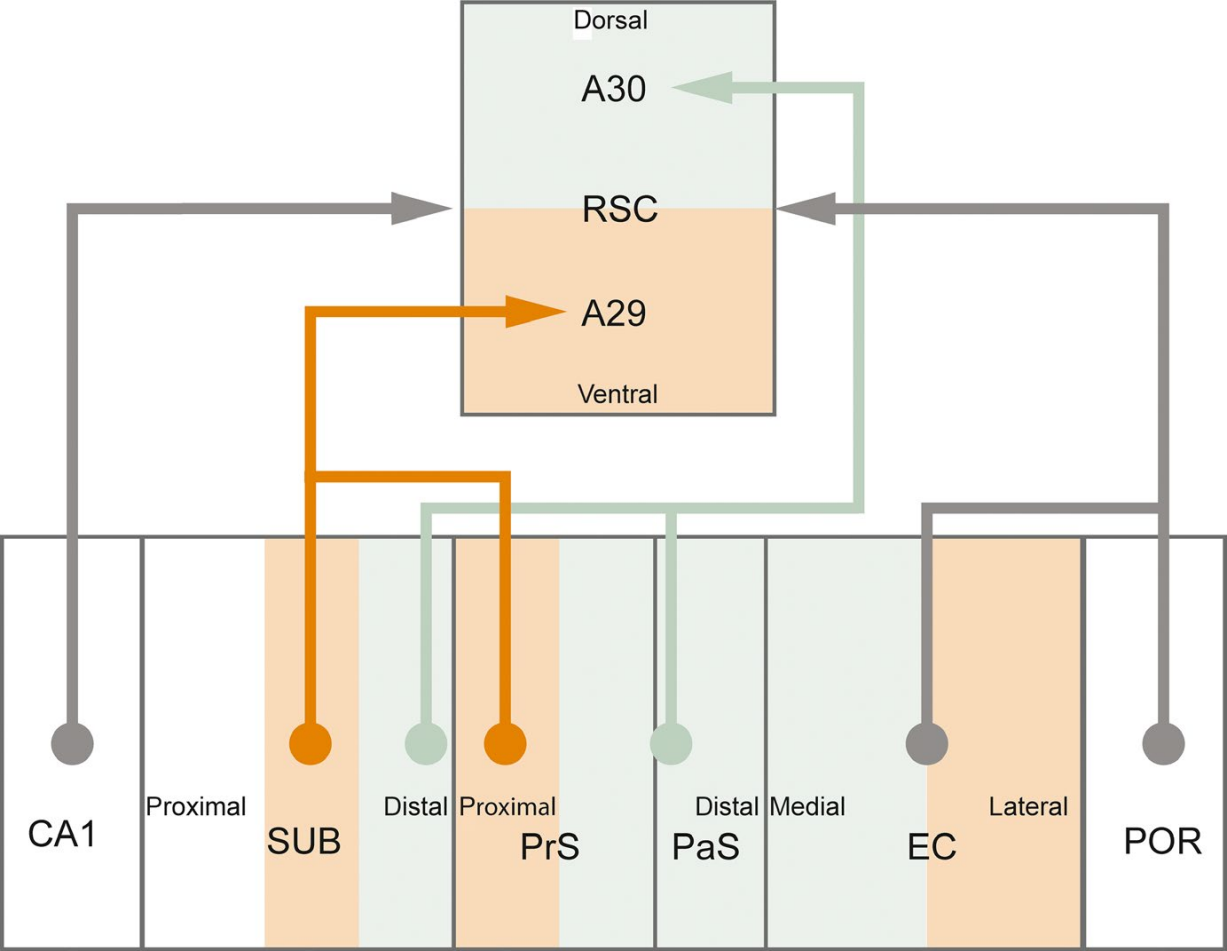
Summary of projections from hippocampus and parahippocampus to retrosplenial cortex. Retrosplenial cortex (RSC) receives inputs from CA1, subiculum (SUB), presubiculum (PrS), parasubiculum (PaS), entorhinal cortex (EC), and postrhinal cortex (POR). These projections are topographically organized such that A30 receives inputs from distal SUB, distal PrS, and PAS, whereas A29 receives inputs from more proximal parts of SUB and proximal PrS. Next, rostral parts of A30 receives inputs only from dorsal parts of SUB, PrS, PaS, EC, and POR, whereas caudal A30 receives inputs also from more ventral parts of these structures (not illustrated in figure). [Colour figure can be viewed at wileyonlinelibrary.com]

Most of our data obtained in postnatal rats are in line with previous studies in the adult rat, showing that the distal half of SUB is the main origin of HF‐PHR outputs terminating in RSC (Kim & Spruston, 2012; Kinnavane, Vann, Nelson, O'Mara, & Aggleton, 2018; Witter, Ostendorf, & Groenewegen, 1990). Additional projections arise from POR, PrS, PaS, and MEC (Figure 11¸ Honda et al., 2011; Ding, 2013). Our data further confirm that the majority of the latter projections arise in the superficial parts of layer V (van Groen & Wyss, 2003; Honda et al., 2011; Insausti, Herrero, & Witter, 1997; Surmeli et al., 2016; Vogt & Miller, 1983), with a minor origin in deep parts of layer VI and superficial layers. Additional very minor projections arise from LEC, PER, and CA1. The latter probably includes a previously reported projection of GABAergic neurons to RSC (Miyashita & Rockland, 2007).

In accordance with earlier published data in both adult rats and monkeys, we show that also in the rat pup, the HF‐PHR projections to RSC are topographically organized along the dorsoventral axis of HF‐PHR. Projections from SUB and PrS are organized such that caudodorsal RSC receives projections from the entire dorsoventral SUB and PrS, whereas rostral and ventral RSC receives afferents only from neurons in dorsal SUB and dorsal PrS, both in pups (present study), as in adults (Aggleton, Wright, Vann, & Saunders, 2012; van Groen & Wyss, 1990, 2003; Wyss & Van Groen, 1992). Our results add to these findings that the projections from PaS are similarly organized, and we therefore expect this to be true in the adult.

We further report that the projections originating in SUB, PrS, and PaS are topographically organized along the transverse plane of HF‐PHR (Figure 11). Similar to what has been reported in a recent paper in adult animals, we observed that in pups, distal SUB projects to dorsal RSC (A30), whereas more proximal parts of SUB project to ventral RSC (A29; Honda & Ishizuka, 2015). Our findings add to these results, showing that distal PrS and PaS preferentially project to A30, whereas more proximal PrS (but not PaS) preferentially projects to A29. The transverse organization of SUB projections to RSC is interesting as proximal SUB neurons are reported to be less spatially modulated than distal ones, suggesting a functional gradient along the proximodistal axis of SUB (Sharp & Green, 1994). In addition, distal neurons in SUB project to areas which are known to be important in processing of spatial information such as MEC, PrS, POR, and the anterior complex of the thalamus, whereas more proximal parts of SUB project to areas known be involved in complex cortical integration such as infralimbic and PER (Witter et al., 1990). The anatomical organization of SUB projections to RSC and the functional gradient within SUB thus suggest a functional difference between A30 and A29. This is in line with previous behavioral studies in which the effect of selective lesions of the two areas of RSC have been assessed (van Groen, Kadish, & Wyss, 2004; Pothuizen, Davies, Aggleton, & Vann, 2010; Pothuizen, Davies, Albasser, Aggleton, & Vann, 2009; Vann & Aggleton, 2005).

Based on both the retrograde and anterograde experiments, we conclude that in pups, like in adults, there is a sparse projection from MEC to RSC. This projection is organized such that rostral parts of A30 receive input from dorsal MEC, whereas caudal parts of A30 receive input from both dorsal and intermediate dorsoventral levels of MEC. We observed an additional gradient in that caudal A30 receives input from neurons located in more medial parts of MEC than those that project to rostral A30. A similar organization of the projections to A29 was not clear in our material. To our knowledge, the topographical organization of MEC inputs to A30 has not been reported previously. This organization is partly mirrored by the reciprocal projection from RSC to MEC in pups, as rostral A29 and A30 project to dorsal parts of EC, whereas caudal A29 and A30 project to more ventral parts of MEC (Sugar & Witter, 2016).

Our temporal analysis shows that all of the individual HF‐PHR projections to caudal RSC are present at P1, although shortly after birth the projections are still weakly developed. During the first postnatal week, the substantial increase in the number of retrogradely labeled neurons indicates a rapid development, such that the projection reaches adult‐like densities between P5 and P10, which is in line with the reported anterograde tracing observations. In contrast, we suggest that projections to rostral RSC develop a few days later than the projections to caudal RSC, as we did not observe retrogradely labeled neurons in HF‐PHR in animals younger than P3 with injections in rostral RSC. We therefore conclude that HF‐PHR projections to RSC are present very early during postnatal development and that the first arriving axons already adhere to the topographic and laminar patterning seen in the adult. This notion is similar to what has been reported in other developing circuits within HF‐PHR and also to what we reported for the reciprocal projection from RSC to HF‐PHR (Canto et al., 2011; O'Reilly et al., 2013, 2014; Sugar & Witter, 2016). All together, these findings suggest that long‐range inputs and outputs with RSC and intrinsic connections of the HF‐PHR system display adult‐like features long before eye‐opening and before the animal starts active exploration of the environment by approximately P15.

## CONFLICT OF INTEREST

The authors report no competing interests.

## AUTHOR CONTRIBUTIONS

All authors designed research. K.G.H. and J.S. conducted experiments and analyzed data. All authors wrote the manuscript.

## Data Availability

Original data will be available upon request. Request should be addressed to Menno P. Witter (menno.witter@ntnu.no).
