# Supplementary material for: Development and topographical organization of projections from the hippocampus and parahippocampus to the retrosplenial cortex
Source: Eur J Neurosci. 2019 Mar 25;50(1):1799–819. doi: 10.1111/ejn.14395 (PMC6767700; doi:10.1111/ejn.14395)
Supplement: Supplementary file 1 [file EJN-50-1799-s001.pdf]

EJN European Journal of Neuroscience FENS Federation of European Neuroscience Societies

Development and topographical organization of projections from the hippocampus and parahippocampus to the retrosplenial cortex

Witter, Menno; Haugland, Kamilla; Sugar, Jørgen

Review timeline:

Submission date: 05-Sep-2018
Editorial Decision: Major Revision
Revision Received: 30-Jan-2019
Accepted: 15-Feb-2019

Editor: Yoland Smith
Reviewer 1: Kathleen Rockland
Reviewer 2: Ricardo Insausti

1st Editorial Decision 05-Oct-2018

Dear Menno,

Your manuscript has been reviewed by two external reviewers as well as by the Section Editor, Dr. Yoland Smith, and ourselves.

The reviews collectively indicate that your experiments generated new and important information. However, there are several issues that need to be clarified/resolved before we can consider your manuscript further for publication in EJN.

Both reviewers found significant merit in your submission and noticed the importance of the work in the field. In general, they considered that the design of experiments and quality of data were appropriate, though one of the reviewers raised some important issues that will have to be considered in the revised manuscript. Some of the key points you should particularly pay attention to are as follows. (1) Provide a more detailed account of the method used to map the localization of labeled cells and discuss the potential limitations of your approach. (2) The presentation of a more quantitative assessment of the density of labeled cells would strengthen the manuscript. (4) Consider the suggestion made by the reviewer about the title and decide if it should be revised or not. (5) Better illustrations of the tracer injection sites are needed. (6) Make sure colors are easy to differentiate from each other in the figures. (7) Try improve the visualization of labeled cells and fibers in figures 2-6. Revise the legends to make sure all elements of the figures are clearly explained. (8) Consider adding a summary diagram of your main findings.

We also note that you have included a reference in the abstract, which we do not encourage. Having said that, over 1000 words came from that publication so please make sure it is cited in the methods section.

When revising the manuscript, please embolden or underline major changes to the text so they are easily identifiable and please don't leave 'track change' formatting marks in your paper. Please ensure that you provide a text and a figure file for the Graphical Abstract (as detailed in the instructions below). When carrying out your revisions please refer to the checklist below and visit the EJN author guidelines at www.ejneuroscience.org

When finalized, please upload your complete revised manuscript onto the website, as a Word file (.doc, or

.docx). Please also ensure that a complete set of tables and figures is included as separate files, even if these have not changed from the originals. At this stage it is necessary to provide high resolution figures. Please see important instructions below.

Please go into https://mc.manuscriptcentral.com/ejn - Author Centre - manuscripts with decisions where you will find a 'create a revision' link under 'actions'. We ask that you please indicate the way in which you have responded to the points raised by the Editors and Reviewers in a letter. Please upload this response letter as a separate Word (.doc or PDF) file using the file designation "Authors' Response to Reviewers" when uploading your manuscript files. Please DO NOT submit your revised manuscript as a new one. Also, please note that only the Author who submitted the original version of the manuscript should submit a revised version.

If you are able to respond fully to the points raised, we would be pleased to receive a revision of your paper within 12 weeks.

Thank you for submitting your work to EJN.

Best wishes,

Paul & John
co-Editors in Chief, EJN

Reviews:

Reviewer: 1

Comments to the Author
This is a technically solid and interesting report on the developmental time schedule of projections from the HF-parahippocampal region to GRS in the rat. I have only positive comments on the experimental design, results, and discussion. For improvements, I have some suggestions for the figures, English usage, and references (see below).

Methods:
I found myself wanting to know how many rats of each gender; i.e. (female, n=X) and male (n=X) , OR equal numbers of…..

“Waltham, MA) and 580/605” This must be “Fluospheres 580/605” but I had to guess that. Please add “fluospheres”

“or Hamilton syringes”: To match the overall level of detail, please give specifics (5µl syringe?) (tip diameter?)

“Anterograde tracers were injected as previously described (O'Reilly et al., 2013).”
This sentence seems out of place. It is essentially repeated a few lines later, at the bottom of the page.

Unclear: “As post-surgery analgesic, animals received carprofen during surgery”

EJN European Journal of Neuroscience FENS Federation of European Neuroscience Societies

“We euthanized the animals 18-30 hours after surgery”: Is this really for BOTH the retrograde and anterograde injections? Isn’t a longer time preferable for BDA injections?

“The thorax was opened with a small-sized scissor and cold Ringer’s solution was transcardially” OK, but better to say that you inserted in the left ventricle and snipped the right auricle (to match the overall level of detail).

“When the liver turned pale,”: 2-5 minutes??

“In case of P0-P2” In the case

“First, the sections were dehydrated in increasing ethanol solutions (50%, 70%, 80%, 90%, 100%, 100%, 100%) followed by two minutes in xylene (VWR) to clear the sections. The sections were then rehydrated in decreasing ethanol solutions
(opposite order as the dehydration protocol) and thereafter placed in cresyl violet solution for” This seems excessive detail! You might just give % of cresyl violet and the immersion time that you used.

“For this purpose, we took the advantage” English: delete “the”

Results
“in the molecular layer of the dorsal extreme of CA1 (Fig. 2A-C)” Do you mean anterior HF??

Subiculum: laminar location of retrogradely labeled neurons = ??

References:

Add T. Miyashita and Rockalnd, 2007: CA1 (inhibitory interneurons) to GRS
Y. Honda….Kaneko (2011) about rat presubiculum
S.L. Ding in nonhuman primate

Figures: The color code is not reader friendly. Can the Authors consider a different color scheme?

English
Abstract: line 7 “as previously reported for intrinsic….” And delte next line “as published previously….”
Simplify last 2 lines “and the projections of RSC to HF-PHR.”
Page 4 “starts to develop around birth, and”: start to develop….
Page 11 “Some injections also resulted in additionally retrogradely” also is sufficient
Page 12: Be consistent about EC; MEC; LEC (here and thoroughout)
Page 14 “These injections, which involved both A29 and A30, were performed in animals older than P10.” : were from animals….
Page 15 “We analyzed anterograde experiments in three of the main input areas, SUB, MEC and PrS.” “anterograde results from injections in three….”
Page 18 “Our temporal analysis shows that all of the individual HF-PHR projections to caudal RSC are present at P1, be it that shortly after birth” Change “be it that” to “although shortly….”

EJN European Journal of Neuroscience FENS Federation of European Neuroscience Societies

Reviewer: 2

Comments to the Author
The manuscript submitted with the title "Development and topographical organization of projections from the (para)hippocampus to the retrosplenial cortex ", is a very interesting topic that addresses the question of the simultaneous maturation of hippocampal formation fields as well as the peripheral cortical adjacent regions (parahippocampal region, PHR).
The manuscript has quality in terms of the neuroanatomical tracing techniques. The study is carefully conducted, but it is doubtful whether the study was conducted with a direct analysis or if it was only the scanned images what made the bulk of the analysis, which has the caveat of missing faint cells the human eye can detect better than an automatic scanning process. The authors should address this issue and discuss the limitations of the analysis method. It would be helpful to have an assessment of the density of the retrograde labeling in the form of tables or histograms according to the injection sites.
However, several problems can be found scattered among different parts of the manuscript.
1) The title is puzzling and misleading in the use of the term "(para)hippocampus". It is rather hippocampal formation (hippocampus plus entorhinal cortex, as they form a connectional unity) plus parahippocampal region. Otherwise the oversimplification of the hippocampal anatomy and related areas, may lead to wrong assumptions, although the need of simplification can be understood in many authors which do not spend enough time in the clarification of the terms. Authors should expand the name of "(para)hippocampus".
2) Some names could be adapted to the neuroanatomical a nomenclature (i.e. p.9, 2nd paragraph, "…..and/or in the molecular layer of the dorsal extreme of CA1", one has to assume that it is the stratum lacunosum-moleculare, rather than the molecular layer of CA1.
3) Figures. It is absolutely necessary to show the size and location of tracer deposits, both anterograde and retrograde to understand the range of the experiments. Overall, the text of the captions is too long, probably due to the topographical complexity of the patterns of labeling.
4) Figure 1. Define the term "paracaudal" in the projection of A3. Please use other colors for tracer deposits; they are difficult to differentiate. Panel C needs further explanatory statements other than the referral to Sugar and Witter (2016).
5) Figure 2. The interpretation of the figure cannot be easily understood with the representation system. Likewise, the color of the dots is not appropriate to allow the differentiation of the different groups. There are white lines which have not been mentioned in the caption. The low-power pictures are almost impossible to see, even to a trained eye. The light is too dim, and it would be more appropriate to represent the labeling as a chart.
6) Figures 3-6 The representation in a flat map presents the same problem of orientation and color mentioned above. In C, the small size of the different tracer deposits representation makes impossible the transmission of the information.
7) Figure 8. It would be nice to see, side by side, the size of the deposit and the adjacent Nissl stained adjacent section.
8) Figure 9. All the panels are too small to appreciate the details of the topography.
9) A summary diagram would be of great help due to the complexity of the study.

Authors' Response 30-Jan-2019

Replies to reviewer's comments

Reviewer: 1

Methods:
I found myself wanting to know how many rats of each gender; i.e. (female, n=X) and male (n=X) , OR equal numbers of…..

We inserted these numbers in the first paragraph of result section (third line). Since in the methods we describe all animals used for surgery, but we only analyzed data from those with successful injections in the correct place, we deemed it more relevant to enter this in the result section.

"Waltham, MA) and 580/605" This must be "Fluospheres 580/605" but I had to guess that. Please add "fluospheres"

Thank you for noticing and we changed accordingly.

"or Hamilton syringes": To match the overall level of detail, please give specifics (5µl syringe?) (tip diameter?)

We provided information about diameter and gauge.

"Anterograde tracers were injected as previously described (O'Reilly et al., 2013)."
This sentence seems out of place. It is essentially repeated a few lines later, at the bottom of the page.

We have deleted the sentence.

Unclear: "As post-surgery analgesic, animals received carprofen during surgery"

We rewrote this sentence; it now reads: Animals received carprofen during surgery, which acted as an analgesic during and the first 24 hours after surgery.

"We euthanized the animals 18-30 hours after surgery": Is this really for BOTH the retrograde and anterograde injections? Isn't a longer time preferable for BDA injections?

Yes, this is correct. BDA does not need more time in pups. Possibly because of short distance needed to travel in small brains. We described and validated this approach in pups in detail in a previous paper (O'Reilly et al 2013).

"The thorax was opened with a small-sized scissor and cold Ringer's solution was transcardially" OK, but better to say that you inserted in the left ventricle and snipped the right auricle (to match the overall level of detail).

We rewrote this sentence to: The thorax was opened with a small-sized scissor and cold Ringer's solution was perfused through the body by inserting the needle in the left ventricle and opening the right auricle.

"When the liver turned pale,": 2-5 minutes??

We have made this more explicit by inserting the time this takes on average: When the liver turned pale (~30 seconds), the perfusion…..

"In case of P0-P2" In the case

We changed accordingly.

"First, the sections were dehydrated in increasing ethanol solutions (50%, 70%, 80%, 90%, 100%, 100%, 100%) followed by two minutes in xylene (VWR) to clear the sections. The sections were then rehydrated in decreasing ethanol solutions
(opposite order as the dehydration protocol) and thereafter placed in cresyl violet solution for" This seems excessive detail! You might just give % of cresyl violet and the immersion time that you used.

We simplified this as suggested. The sentence now reads: The sections were dehydrated in ethanol, cleared in xylene (VWR) and rehydrated before placed in cresyl violet (0.1% in water, C5042, Sigma) for two to six minutes.

"For this purpose, we took the advantage" English: delete "the"

We changed accordingly.

Results
"in the molecular layer of the dorsal extreme of CA1 (Fig. 2A-C)" Do you mean anterior HF??

This is an important point and we realize that our way of describing is unclear. However, the suggested formulation also may result in confusion in view of the nomenclature of the primate brain, where the anterior HF is commonly used as to refer to the temporal part of HF. We have changed this throughout the manuscript with ' the anterior part of dorsal CA1'.

Subiculum: laminar location of retrogradely labeled neurons = ??

We rephrased the first sentence, adding pyramidal layer: All injections resulting in retrogradely labeled neurons within HF-PHR, displayed retrogradely labeled neurons in the pyramidal layer of SUB.

References:

Add T. Miyashita and Rockalnd, 2007: CA1 (inhibitory interneurons) to GRS
Y. Honda….Kaneko (2011) about rat presubiculum
S.L. Ding in nonhuman primate

We added the suggested references in discussion and thank the reviewer for suggesting these additions. The sentences in the discussion now read:

Additional very minor projections arise from LEC, PER and CA1. The latter probably includes a previously reported projection of GABAergic neurons to RSC (Miyashita and Rockland, 2007).

Additional projections arise from POR, PrS, PaS and MEC (Honda et al., 2011; Ding 2013).

Figures: The color code is not reader friendly. Can the Authors consider a different color scheme?

We have changed the color code of the flatmaps, realizing that the colors might be difficult to differentiate. We do ask understanding however that we put a lot of information in each figure and we tried to keep colors stable throughout the manuscript. We carefully checked that all colors are explicitly

mentioned in the legends and we have increased the size of the most relevant flatmaps to increase visibility.

English

Abstract: line 7 “as previously reported for intrinsic….” And delete next line “as published previously….”

Changed accordingly and we also deleted the reference as well as others throughout the Abstract as requested by the editors.

Simplify last 2 lines “and the projections of RSC to HF-PHR.”

Changed accordingly.

Page 4 “starts to develop around birth, and”: start to develop….

Changed accordingly.

Page 11 “Some injections also resulted in additionally retrogradely” also is sufficient

Changed accordingly.

Page 12: Be consistent about EC; MEC; LEC (here and throughout)

In the introduction of the paper we use EC as well as LEC and MEC to introduce the two subdivisions. In the results we initially used EC in all statements that hold true for both LEC and MEC. In the revised version we replaced ‘in EC’ with ‘in both LEC and MEC’

Page 14 “These injections, which involved both A29 and A30, were performed in animals older than P10.” : were from animals….

Changed accordingly.

Page 15 “We analyzed anterograde experiments in three of the main input areas, SUB, MEC and PrS.” “anterograde results from injections in three….”

Changed accordingly.

Page 18 “Our temporal analysis shows that all of the individual HF-PHR projections to caudal RSC are present at P1, be it that shortly after birth” Change “be it that” to “although shortly….”

Changed accordingly.

EJN European Journal of Neuroscience FENS Federation of European Neuroscience Societies

Reviewer: 2

The study is carefully conducted, but it is doubtful whether the study was conducted with a direct analysis or if it was only the scanned images what made the bulk of the analysis, which has the caveat of missing faint cells the human eye can detect better than an automatic scanning process. The authors should address this issue and discuss the limitations of the analysis method.

We inspected all tissue in the microscope. We feel that there might be a misunderstanding, in that we did not automatically analysed the data. We obtained digital images with the use of automated scanners. Next, two independent persons (K.G.H and J.S.) assessed the digital sections and recorded the fine position of labeled neurons and injection sites; thus the position and number of labeled neurons was not automatically detected, but manually, based on digital images. The digital images were obtained with a 20X objective and were in general of very good quality. Please see for instance CA1 neurons in Fig 3 (originally figure 2), which are weakly labeled compared to subicular neurons, but which are clearly visible compared to unlabeled neurons.

In the few cases when there were discrepancies between the two observers we returned to a direct assessment to resolve any discrepancies. We have updated the methods section so that this procedure is more clearly described.

It would be helpful to have an assessment of the density of the retrograde labeling in the form of tables or histograms according to the injection sites.

We have included a table providing a semiquantitative representation of the data.

The title is puzzling and misleading in the use of the term "(para)hippocampus". It is rather hippocampal formation (hippocampus plus entorhinal cortex, as they form a connectional unity) plus parahippocampal region. Otherwise the oversimplification of the hippocampal anatomy and related areas, may lead to wrong assumptions, although the need of simplification can be understood in many authors which do not spend enough time in the clarification of the terms. Authors should expand the name of "(para)hippocampus".

We appreciate the comment and agree that we were likely guided a bit too much by the aim to have a compact title. We changed the title to "Development and topographical organization of projections from the hippocampus and parahippocampus to the retrosplenial cortex". We are aware that some colleagues prefer the nomenclature proposed by the reviewer, but we take the liberty to include the entorhinal cortex in the parahippocampal region, as we do in all our publications.

Some names could be adapted to the neuroanatomical a nomenclature (i.e. p.9, 2nd paragraph, "…..and/or in the molecular layer of the dorsal extreme of CA1", one has to assume that it is the stratum lacunosum-moleculare, rather than the molecular layer of CA1.

We made changes accordingly throughout the manuscript, including legends where appropriate.

Figures. It is absolutely necessary to show the size and location of tracer deposits, both anterograde and retrograde to understand the range of the experiments. Overall, the text of the captions is too long, probably due to the topographical complexity of the patterns of labeling.

We added a new figure 2 showing the actual histology of the cases used in the figures in the paper, in conjunction with their location in the flatmaps. Moreover, for all analyzed cases, in the new table 1 we provide the volume and size of the injections.

EJN European Journal of Neuroscience FENS Federation of European Neuroscience Societies

Figure 1. Define the term “paracaudal” in the projection of A3. Please use other colors for tracer deposits; they are difficult to differentiate. Panel C needs further explanatory statements other than the referral to Sugar and Witter (2016).

We thank the reviewer for the comments and changed “paracaudal” to “caudal”.

As replied to reviewer 1, we aimed to make a consistent use of colors in all figures. Although we appreciate that all individual injections cannot be easily differentiated in Figure 1A, we decided against changing the colors. The purpose of the figure, at this magnification, is not to provide details for all injections, but to convey the important message that we covered a substantial part, if not all of RSC with our injections. We feel that the subsequent representation of the injections in the new Figure 2, the individual flatmaps and the data provided in table 1 is sufficient.

We have added a new paragraph to the methods explaining the transitions from fig A to B to C.

Figure 2. The interpretation of the figure cannot be easily understood with the representation system. Likewise, the color of the dots is not appropriate to allow the differentiation of the different groups. There are white lines which have not been mentioned in the caption. The low-power pictures are almost impossible to see, even to a trained eye. The light is too dim, and it would be more appropriate to represent the labeling as a chart.

We agree with the reviewer that it might be hard to retrieve the relevant details from the figure. We have changed the layout so that the figure is hopefully easier to follow. Explanation of the white lines have also been added to the caption. We have changed the color code of the flatmaps for improved visibility. We have also done graphical modifications of the images to improve visibility. If the reviewer do not find that the changes have improved the figure enough we will change the low power images into a chart. However, we find it important to display images of the “raw” data in addition to the flatmaps and the added semiquantitative data in the new Table 1.

Figures 3-6 The representation in a flat map presents the same problem of orientation and color mentioned above. In C, the small size of the different tracer deposits representation makes impossible the transmission of the information.

We have changed the color code of the flatmaps. We have also improved the layout of the figures to improve the visibility. See our comment regarding figure 2 above.

Figure 8. It would be nice to see, side by side, the size of the deposit and the adjacent Nissl stained adjacent section.

Panels A1, B1, C1 covers this request. These panels include the injections overlaid on the neighboring nissl stained section.

Figure 9. All the panels are too small to appreciate the details of the topography.

We have changed the layout of the figure so that image size is increased.

A summary diagram would be of great help due to the complexity of the study.

We have included a new figure 11 which contains a summary diagram of the main findings which we feel are important to emphasize to our readers
